# Disentangling Subsidence from Shallow Soil Processes and Gas Extraction in a Dutch UNESCO World Heritage Polder with InSAR and Data Assimilation

**DOI:** 10.1007/s41748-025-00885-8

**Published:** 2025-12-02

**Authors:** Manon Verberne, Kay Koster, Hans de Bresser, Peter A. Fokker

**Affiliations:** 1 Faculty of Geosciences, Dept. of Earth Sciences, Utrecht University, Princetonlaan 8a, 3584CB Utrecht, The Netherlands; 2 TNO Geological Survey of the Netheralnds, Princetonlaan 6, 3584CB Utrecht, the Netherlands

**Keywords:** Coastal subsidence, Vertical land motion, InSAR, Cultural heritage, Gas extraction, The Netherlands

## Abstract

Many deltas and coastal plains are densely populated because of their fertile soils and strategic locations. Yet, these areas face substantial subsidence challenges, as a result of human subsurface activities at multiple depths. Subsidence studies so far focus on the effects of individual causes, limiting the understanding of total subsidence. This is also the case in the Netherlands, with much of its coastal land already below mean sea level. This is primarily the result of centuries of water management. The subsurface in these areas is composed of soft soil, making them highly susceptible to subsidence by shrinkage, oxidation, and compaction. The effects of subsidence are locally aggravated by deeper hydrocarbon extraction. This study centers around the Beemster polder, a UNESCO World Heritage site that exemplifies complex human-induced subsidence originating from multiple depths. We distinguish the contributions of the shallow and deep subsidence causes in a joint model. We quantified the contributions and optimized modeling with data assimilation. The results of the combined effects were optimized for 2015–2022 using InSAR data. Locations with the thickest surficial peat layers exhibit over 5 mm/year subsidence, yet the effect of thick clay beds is also substantial. The expected subsidence related to gas extraction is not expected to exceed 30 mm in the period 2020–2050. Findings from this work deepen our understanding of subsidence dynamics, offering an approach that can be applied to similar subsidence-prone coastal and deltaic regions worldwide, where multiple overlapping factors drive subsidence.

**Graphical Abstract:**

This study aims to understand and quantify the contributions of different human-induced subsidence processes in a coastal UNESCO World Heritage polder containing several actively producing gas fields. Subsidence is assessed by integrating observational data with information on (sub)surface characteristics and applying subsidence models. The workflow, summarized in the graphical abstract, involves several steps. First, all input datasets are compiled. InSAR and levelling measurements provide observational subsidence data. Subsurface information includes a lithostratigraphic model of the shallow subsurface, gas production data, and geomechanical properties of the gas reservoirs. Both the shallow model and gas extraction at depth are modeled analytically. These analytical models include parameters that are calibrated using the Ensemble Smoother with Multiple Data Assimilation (ES-MDA). This optimization step enables a quantitative separation of contributions from different subsidence processes. The resulting parameter sets are also used to project future subsidence, based on planned gas extraction and assuming that shallow subsidence continues in line with observed trends. The results reveal distinct spatial patterns in subsidence, particularly highlighting the contrast between areas with intact peat layers and those where peat has been removed historically. Subsidence from shallow processes is related to the presence and thickness of surficial peat and clay deposits. Deep subsidence due to gas extraction completes the overall subsidence pattern. These insights are valuable for guiding mitigation strategies by policymakers and stakeholders. Moreover, the study emphasizes the need for a comprehensive, integrated framework for understanding subsidence processes, particularly in vulnerable coastal and deltaic environments worldwide. 
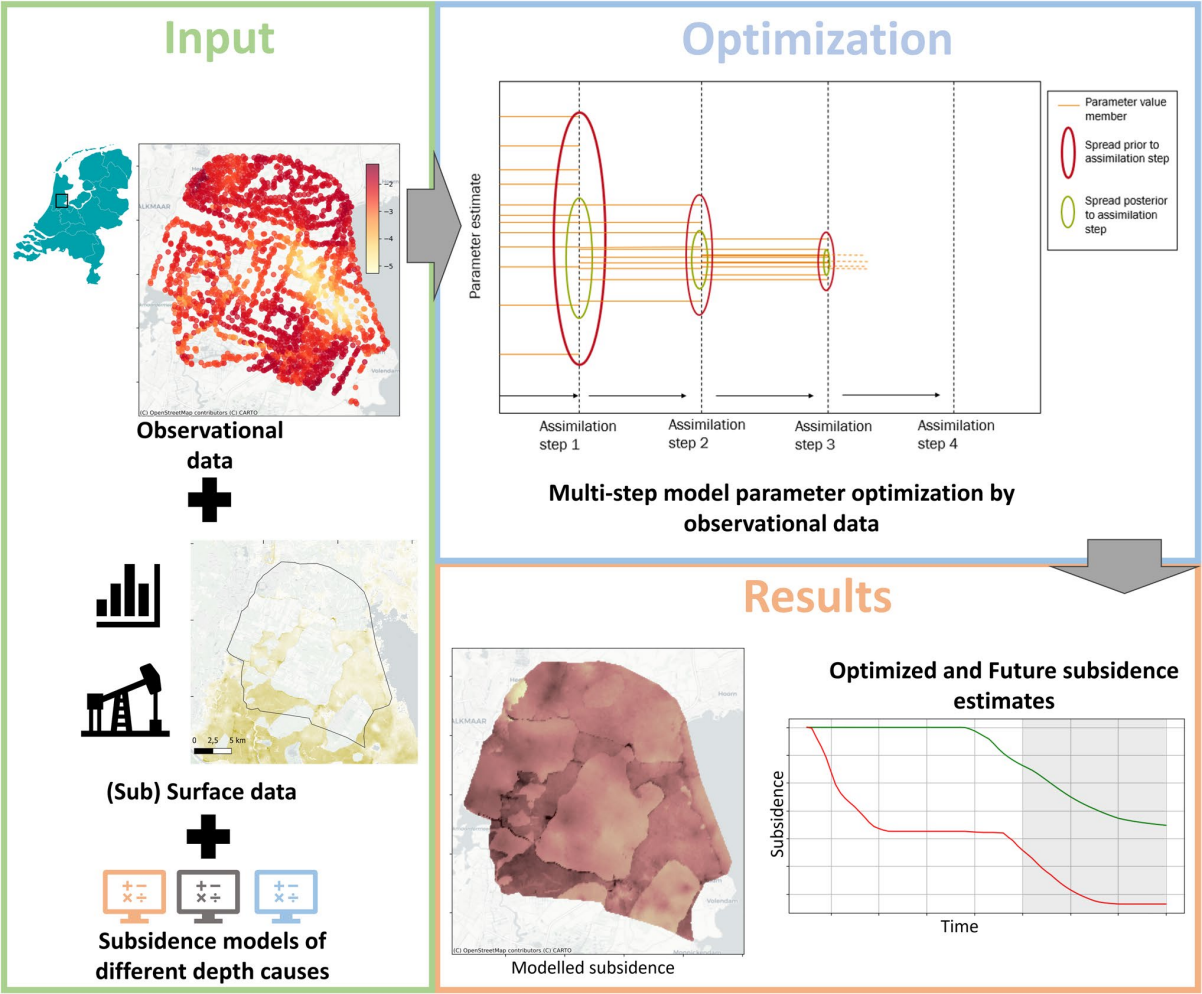

## Highlights

This study underscores the importance of a comprehensive approach that considers the interplay between many factors of human-induced coastal subsidence.

Shallow subsidence is linked to the thickness, lithology, and depositional environment of the coastal deposits.

The highest subsidence rates are linked to surficial-peat-rich areas.

When a gas field with active extraction underlies a peat-rich area, the total subsidence rates reach the maximum estimated values.

## Introduction

Coastal and delta plains rank among the most densely populated regions globally, largely due to their fertile soils and strategic locations (Neumann et al. 2015; Syvitski et al. 2009); these characteristics have historically made them attractive for human settlement and development (Anthony et al. 2024). However, such regions present significant challenges, with subsidence being a primary concern (Shirzaei et al. 2021; Syvitski 2008; 2009). Subsidence-related risks include damage to infrastructure, buildings, and historical sites (Prosperi et al. 2023), as well as threats to ecosystems and an increase of flood risks (Giosan et al. 2014). Ongoing sea-level rise further exacerbates the vulnerability of these low-lying regions (Griggs and Reguero 2021; Magnan 2022; Nicholls 2011)

An increasing number of coastal and deltaic regions around the world are threatened by subsidence (Giosan et al 2014). The Mississippi Delta in the United States (Törnqvist et al. 2008), the Mekong Delta in Vietnam (Minderhoud et al. 2018), and the coastal city of Jakarta (Abidin et al. 2011) serve as prominent examples of areas under threat. Subsidence in these and other vulnerable regions often stems from a combination of natural processes and human activities (Candela and Koster 2022; Chaussard et al. 2013; Tosi et al. 2013). Contributing factors include the natural consolidation of sediments (Zoccarato et al. 2018), the decomposition of organic matter (Van Asselen et al. 2018), compaction by the built environment (Parsons et al. 2023; Zhao et al. 2019), and the extraction of subsurface resources such as groundwater (Galloway and Burbey 2011; Minderhoud et al. 2017), salt (Fokker and Osinga 2018; Fokker et al. 2018), and hydrocarbons (Fibbi et al. 2025; van Eijs and van der Wal 2017). These processes together make subsidence a complex and pervasive challenge for coastal and deltaic areas worldwide. The various processes involved are often still studied and discussed separately, though the need for a multidisciplinary approach is often emphasized (Fibbi et al. 2024; Shirzaei et al. 2021; Candela and Koster 2022). Verberne et al. (2025b) applied a multidisciplinary approach to the area around Ravenna, Italy. Here, the results of the deep model were scaled by a factor and combined with an analytical shallow model in one approach. In this study shallow and deep subsidence are both modelled analytically and optimized in in a single approach.

Subsidence is a particularly pressing issue in the coastal plain of the Netherlands (Fokker et al. 2025). This is due to the country’s low–lying coastal position, the presence of vast, thick, soft soil layers – especially organic-rich – and its extensive history of water management and land reclamation (Verberne et al. 2023). As a result of subsidence, almost half of the countries’ populated coastal plains already lie below mean sea-level (Koster et al. 2018).

**Fig. 1:**
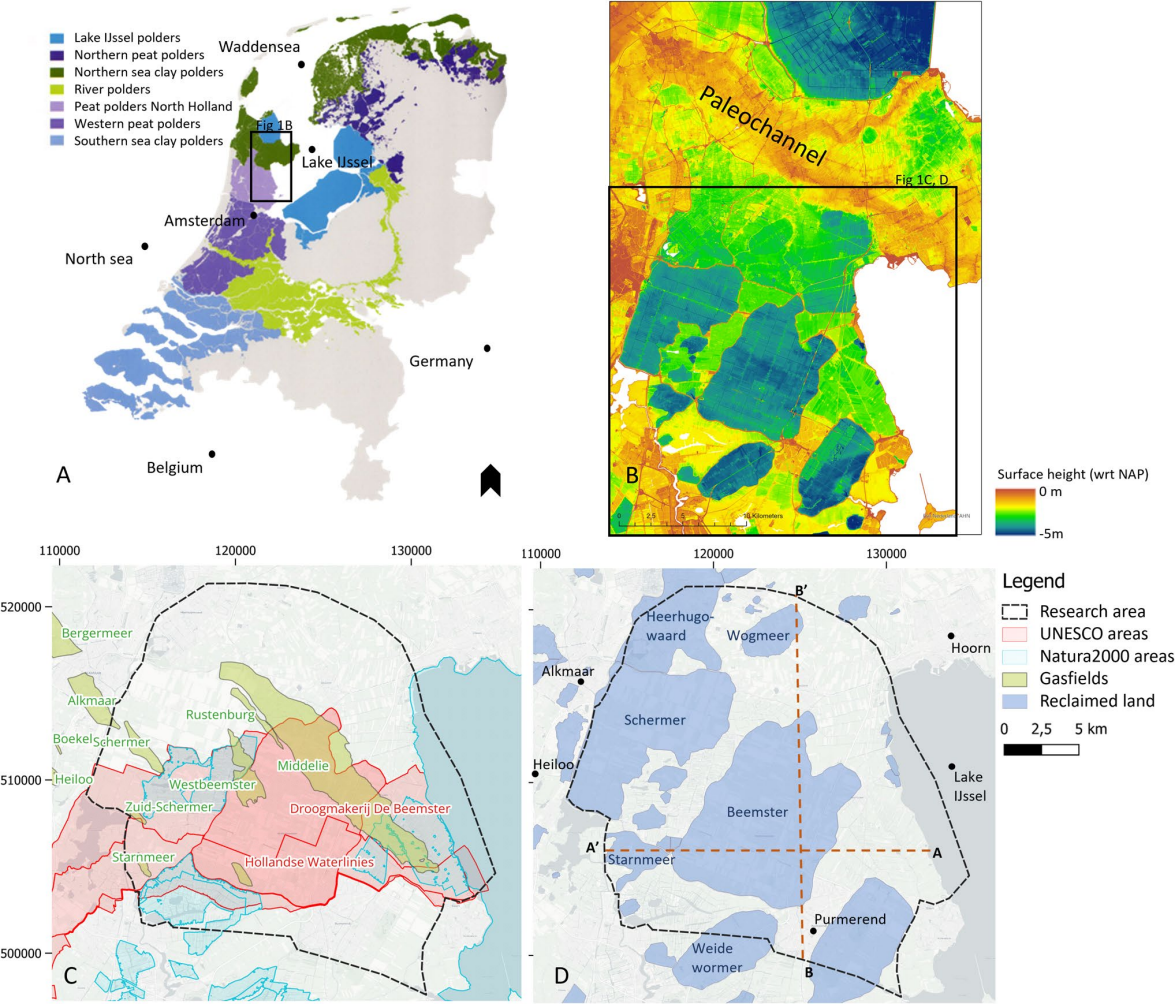
**A** map of the Netherlands showing the areas that accommodate polders. Adjusted from Steenbergen et al. (2009). **B** Zoomed image surrounding the research area projecting the surface level, derived from AHN (2025). **C** Map of the research area in the northwest of the Netherlands. The UNESCO and Natura2000 areas and the gas fields are indicated. Note that both active and inactive gas fields are indicated here, though only the active gas fields will be discussed in terms of subsidence modeling.** D** Reclaimed lands and relevant topographic names in the study area. The geological profiles of Fig. 9 and the location of the subarea (Fig. 6) are indicated

The Netherlands has a long history of water management, as evidenced by the creation of numerous polders, which are stretches of land where groundwater levels are artificially managed. Some polders consist of reclaimed land from the sea or coastal lakes, closed off by dikes (Schultz 1983) (Fig. 1A). While these polders have facilitated extensive agricultural and urban development, they are also highly susceptible to subsidence (Verberne et al. 2023; Fokker et al. 2019). Drainage practices within reclaimed lands and surrounding polders contribute to progressive shrinkage of clay, oxidation of organic materials, and compaction of soft soil (e.g. van Asselen et al. 2018; van Hardeveld et al. 2017). Additionally, the extraction of hydrocarbons has intensified subsidence across the coastal plain (Ketelaar 2009). The Groningen gas field for example, Europe’s largest, has been a significant contributor to coastal subsidence in the northeast of the country in the past 60 years (Van Thienen-Visser and Fokker 2017). Smaller gas fields throughout the Netherlands also play an important role in this ongoing subsidence (Fokker et al. 2012; 2016).

The Beemster polder region (Fig. 1B) in the western coastal zone of the Netherlands is an area where subsidence is the result of multiple human-induced processes, at different depth levels. Disentanglement of the significant subsidence processes is imperative for effective mitigation. The area hosts both UNESCO World Heritage sites and European protected nature areas (Natura2000). The UNESCO World Heritage site ‘Beemster polder’ was reclaimed in the seventeenth century, alongside nearby polders such as the Purmer and Schermer (Hoeksema 2007) (Fig. 1C). These polders are reclaimed coastal lakes that existed as a remnant of bog peat mining. Within these lakes, peat has largely disappeared as a result of mining and later wave-erosion, whereas surrounding the lakes, peat has remained present. The area also covers eight different gas fields (Gee et al. 2016), with active gas extraction from four of them. The resulting subsidence pattern in the region is a complex mosaic, shaped by the interplay of subsurface properties of the soft soil, historical reclamation practices, and ongoing gas extraction activities.

The present study seeks to disentangle the various causes of subsidence in the coastal plain in and around the Beemster polder, with a specific focus on quantifying the contributions of shallow and deep causes. Subsidence modeling is optimized by integrating InSAR data into a data assimilation procedure. This provides a comprehensive understanding of the subsidence dynamics in the region. The findings from this study contribute to SDG 11 (Sustainable Cities and Communities) by enabling more effective management strategies to mitigate the impacts of subsidence and ensure the long-term sustainability and safety of this vulnerable and complex low-lying area. Additionally, the study supports SDG 6 (Clean Water and Sanitation) and SDG 13 (Climate Action) by informing strategies for sustainable groundwater and land-use management. The methodology demonstrated in this research can be replicated in other regions with multiple contributing causes.

### Study Area

The study area is situated between the cities of Purmerend in the south and Hoorn in the north. The area is bounded to the east by lake IJssel and to the west by the municipalities of Heiloo and Alkmaar. Two different depths levels are important for the current subsidence patterns: shallow soft coastal soils and deep gas reservoirs.

**Fig. 2:**
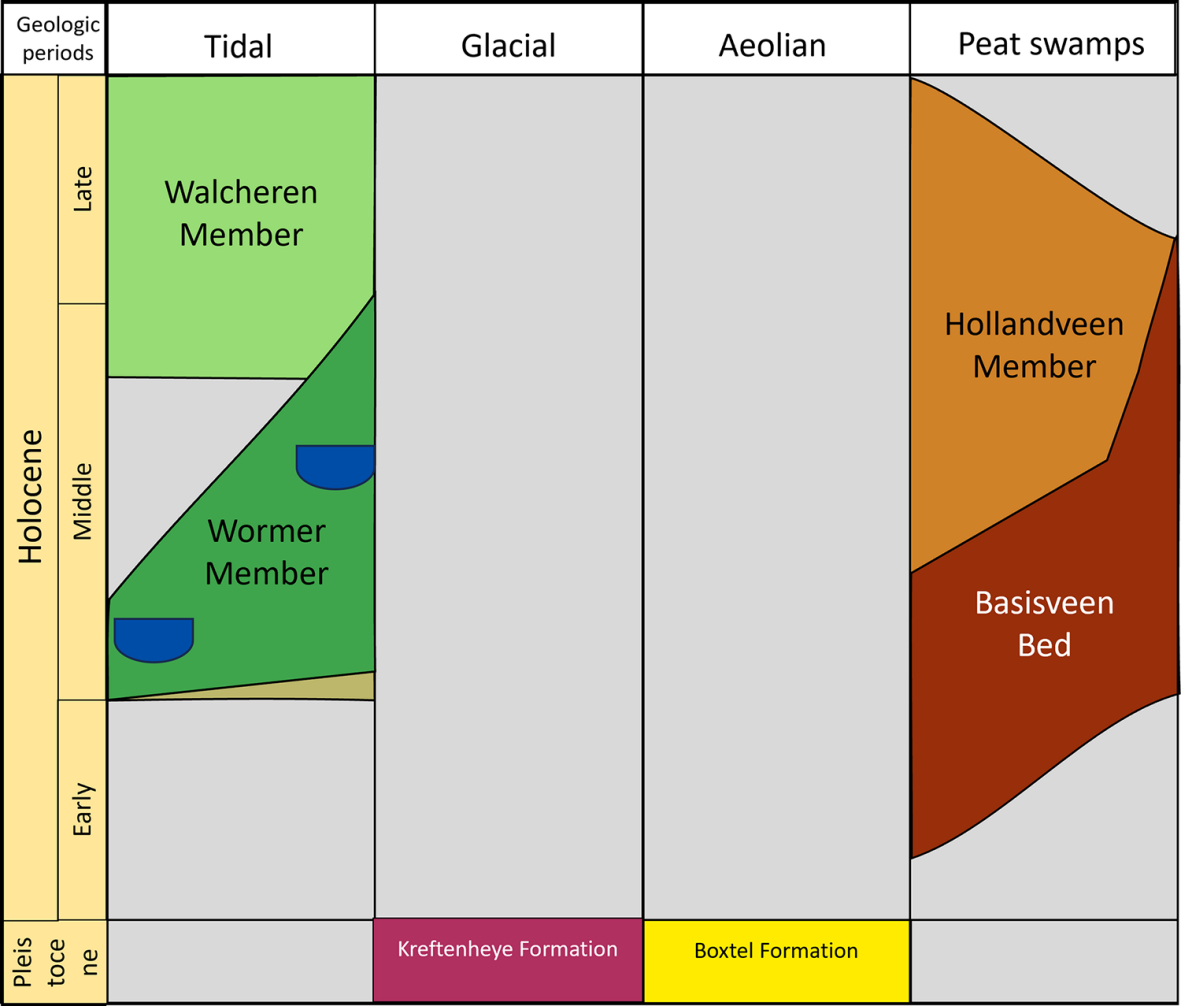
Lithostratigraphic column of the units of Pleistocene and Holocene age in the research area, based on Vos (2015). The columns use the official lithostratigraphic nomenclature of the Netherlands (TNO-GDN 2024). The stratigraphic units are depicted in profile form in Fig. 7. The blue units within the Wormer Member indicate sandy tidal channel formation and the brown refers to the Velsen Bed, a stiff organic-rich clay bed

The lithostratigraphy of coastal geological units relevant for shallow causes of subsidence in the study area is provided in Fig. 2. Underlying the coastal sequence are tens of meters thick deposits of Pleistocene age. These deposits consist of a complex of alternating sandy to clayey marine, fluvial and (peri-)glacial deposits (Peeters et al. 2015; van Aarle et al. 2024). The uppermost lithostratigraphic unit of Pleistocene age (Boxtel Formation), consists of a several meters thick aeolian sand bed (Fig. 7). During the early to mid-Holocene, the groundwater level rose in tandem with post-glacial sea-level rise, forming a basal peat bed on the Boxtel Formation, between ca. 9,500 and 6,000 year BP (Basisveen Bed) (Koster et al. 2017). These peatlands subsequently drowned, and the area transformed into an open tidal basin (Wormer Member) (Vos 2015). The tidal basin deposits consist of alternating sand-clay beds, with local erosion into the underlying basal peat and aeolian Pleistocene sand beds. When around 5,500-year BP eustatic sea-level rise rates decreased, the open tidal basin was closed off by the formation of a beach-barrier, transforming the region into a freshwater swamp where large-scale peat formation was possible (Hollandveen Member) (Beets & van der Spek 2000). Coeval with the onset of peat formation, a tidal channel system existed at the northern fringe of the study area (Walcheren Member, ‘paleochannel’ Fig. 1B), hampering local peat formation by clastic sedimentation, resulting in decreasing peat thickness in northern direction.

Reclamation of the lakes had two purposes: reducing flood risks for the surrounding cities and gaining fertile soil for agricultural purposes to feed the growing city populations. The first land reclaimed in the research area was the Wogmeer polder (1609 CE), followed by the first large polder De Beemster in 1612. The final polder reclaimed in the area was the Schermer in 1635.

The Alkmaar area is the second-largest onshore production area of natural gas in the Netherlands after Groningen (Gee et al. 2016; Van Lith 1983). The gas reservoirs within the study area are situated at depths ranging from 1200 to 2200 m and are part of the Permian Upper Rotliegend Group and Zechstein Group (Table 1).

**Table 1:**
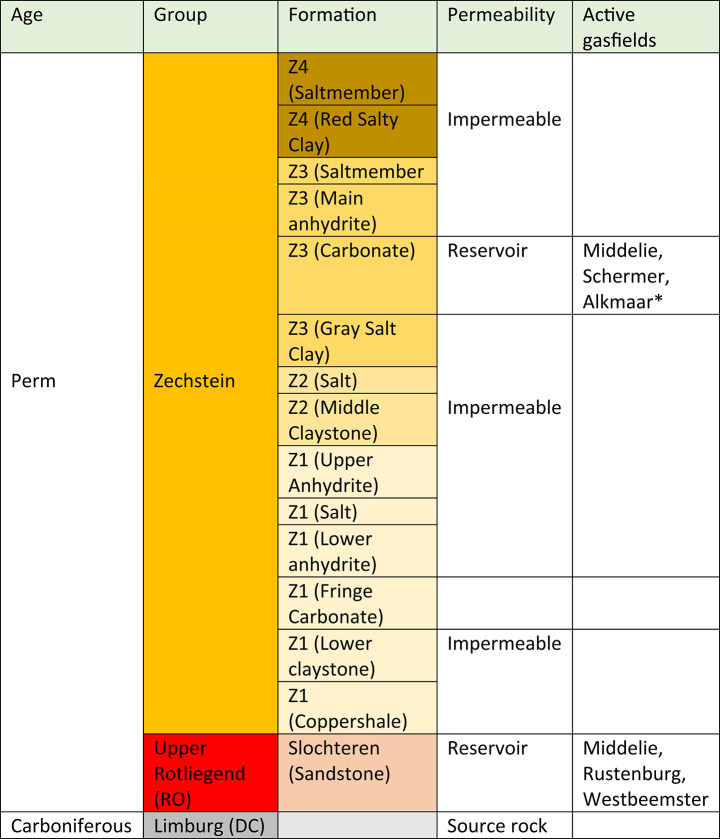
Geological timescale of the formations related to the gas reservoirs for active extraction (source, reservoir and seal)

Note that for Middelieboth Upper Rotliegend (Slochteren) and Zechstein reservoirs are actively producing

^*^The Alkmaar field is currently used for gas storage, not gas extraction

The Zechstein reservoirs were deposited in a northeast-prograding carbonate platform, with an average reservoir thickness of 15 m, though the deposits can reach up to 40 m in thickness locally. They are sealed by overlying Zechstein salt deposits (NAM 2023). The Rotliegend group reservoirs, part of the Slochteren sandstone formation, a 120–240-m-thick unit deposited in a dry continental eolian environment. These reservoirs are also sealed by the overlying Zechstein Group. The source rock for all reservoirs in the area is the coal deposits of the Limburg Group (NAM 2023).

The Zechstein Group comprises two gas-bearing fields with active production in the study area: Middelie and Schermer. The Alkmaar field is currently used for gas storage. The Upper Rotliegend Group contains three gas-bearing reservoirs with active production: Middelie, Rustenburg, and Westbeemster. Note that the Middelie field also has active production from the Zechstein reservoir.

Production began in the 1970s with the Middelie Zechstein and Rotliegend reservoirs. The first production phase, spanning 1975–1991, ceased due to high water production. The second production phase commenced in 2007, restarting operations at the Middelie Zechstein reservoir and initiating production at the Westbeemster Rotliegend. Rustenburg Rotliegend production began in 2009, followed by the Middelie Rotliegend in 2015. Currently, multiple wells are active, with a shared production location for the Westbeemster and Rustenburg reservoirs (NAM 2023). Production in the Bergen concession began in 1992 from the Schermer field and continues today, albeit at significantly reduced levels since 2001 (TAQA 2020).

No significant subsidence has been observed in association with gas extraction from the Schermer field within the study area. Given the minimal production and lack of measurable subsidence in the study area, the contribution of Schermer field to subsidence is deemed negligible and will not be considered further in this study.

## Materials and Methods

**Fig. 3:**
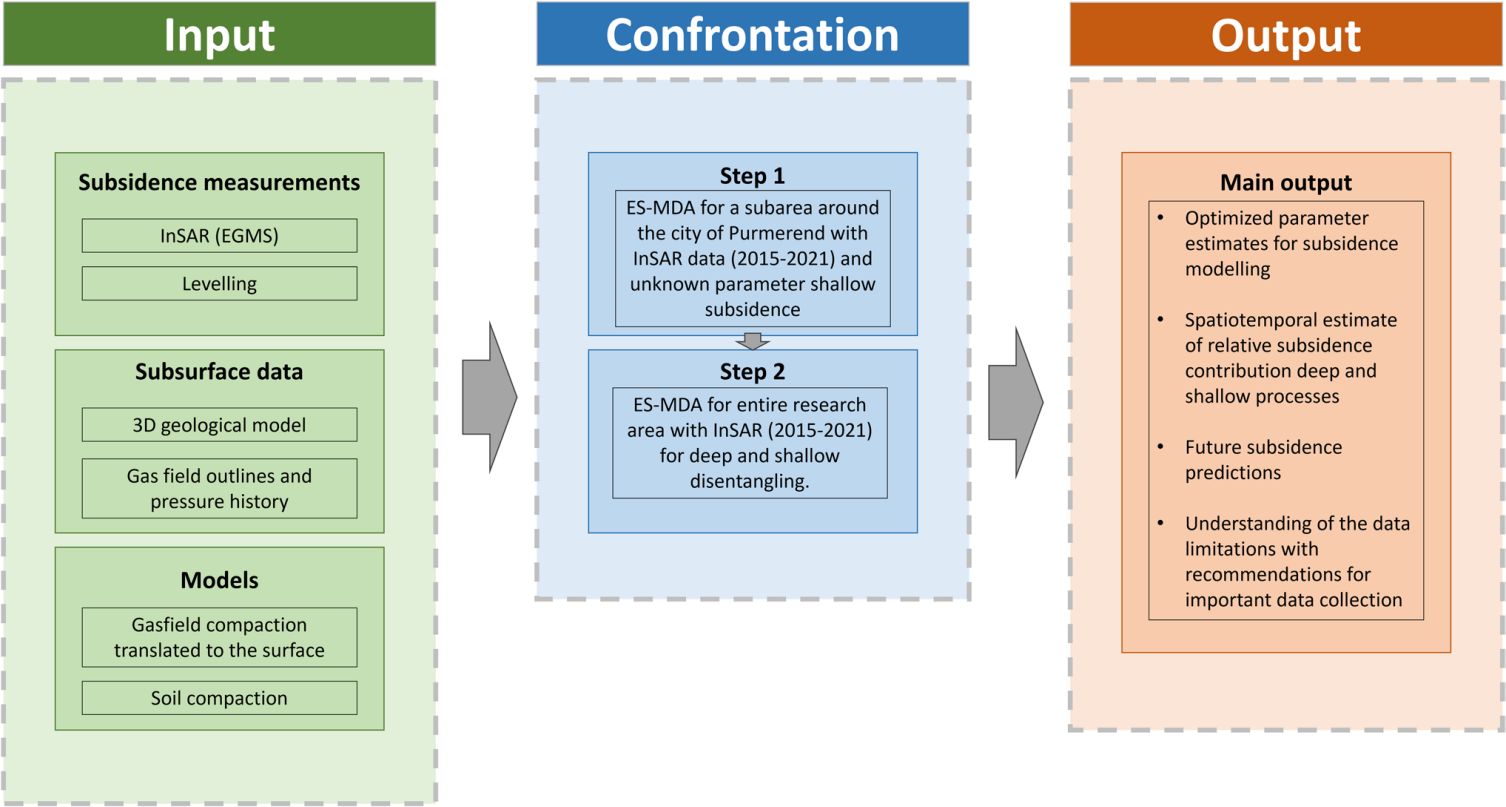
Workflow as applied in this study. First the input data is gathered and combined in the right format for the confrontation step. The confrontation step consists of two sub steps. First parameters for the shallow model are optimized using InSAR data in a subset of the larger area with ES-MDA. In the second step the outcome of the previous step is combined in one ES-MDA with both deep and shallow modelling applied to the entire research area. The main output of this methodology consists of estimates of the parameters, estimates of subsidence in the past from the model, and future predictions of subsidence. This combined indicates the relative and absolute contribution of the different subsidence processes modelled

In this study, various types of input data and models are combined to optimize subsidence parameters through a two-step data assimilation approach (Fig. 3). In this section, first the input data and models used are discussed. Secondly, the confrontation step for optimization is explained. Lastly, the study-specific setting and assumptions are outlined.

### Subsidence Measurements

#### InSAR

Synthetic Aperture Rader Interferometry (InSAR) allows for estimation of small displacements of objects on the surface, using interferometry of radar images (Ferretti et al. 2007). In this study, InSAR information derived from Sentinel-1 is used, available in processed form by the European Ground Motion Service (EGMS 2024a; Calero et al. 2023). The ortho product level of the EGMS is used, which consists of components of motion vertically and horizontally (east–west) resampled to a grid of 100 × 100 m (EGMS 2024b). Details on the fundamental processing steps, a technical summary and the algorithm used to process the InSAR data can be found at EGMS (2024a,b).

**Fig. 4:**
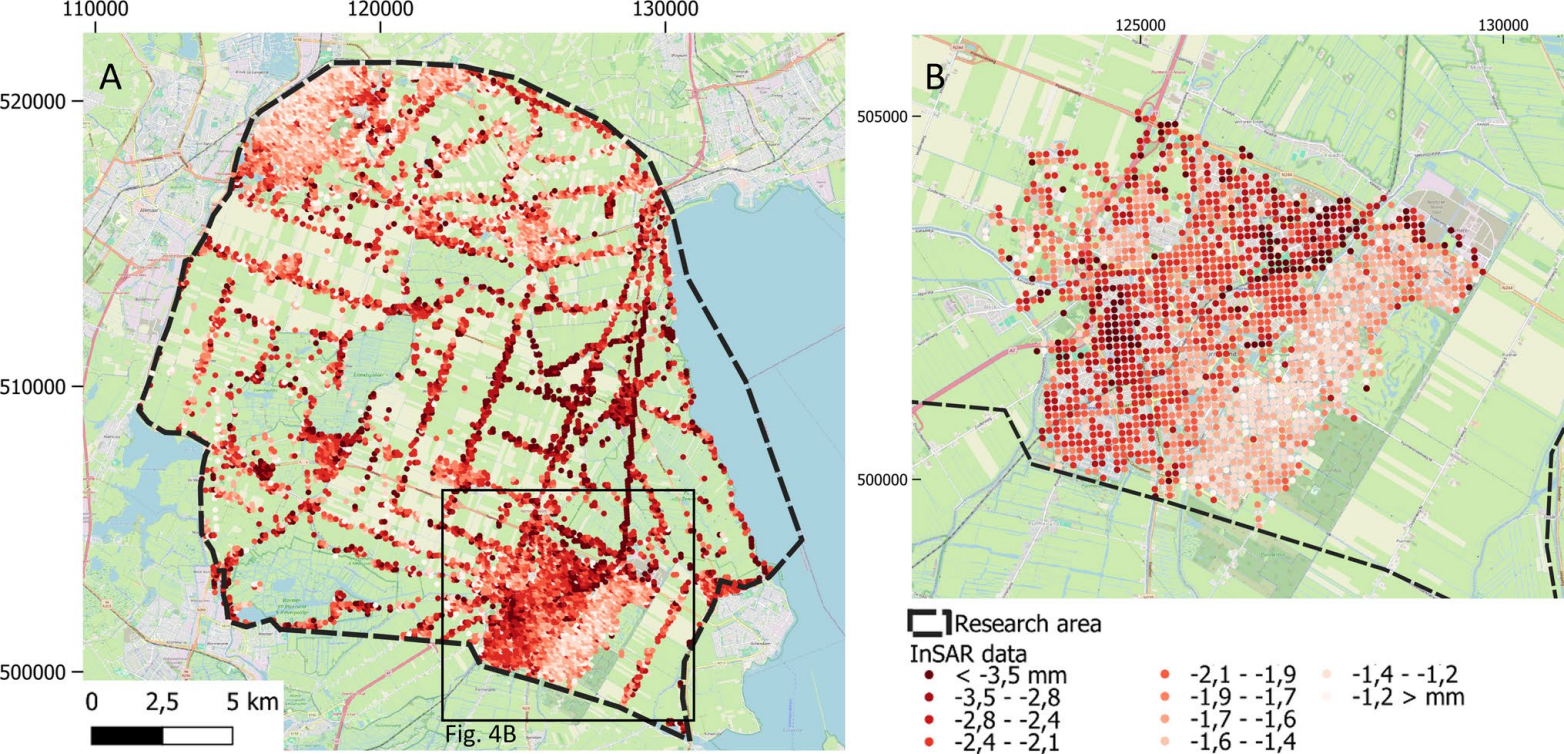
EGMS derived displacement estimates for the vertical direction resampled to a 100 × 100 grid for the total research area (**A**) and the Purmerend subarea (**B**). The coordinate system of the maps is Rijksdriehoek (RD)-new

Figure 4A shows the EGMS data for the research area, for which spatial variation in the subsidence rates is made visible by plotting the linear subsidence rate in millimeter per year over the entire timescale of the available data (2015–2022) Fig. 4B shows the EGMS data selected for the Purmerend subarea.

To prevent overfitting our subsidence models to parts of the area with more datapoints while at the same time maintaining a substantial amount of datapoints, the datapoints are resampled by selecting a random datapoint per 300 × 300 m grid. For the Purmerend subregion data points have not been resampled as the probability of overfitting is considered negligible with the distribution of points being relatively even.

#### Levelling Data

Levelling is a surveying technique in which benchmarks are placed around an area at which elevation measurements are taken, usually every few years. The data contains the elevation of the benchmarks with respect to a datum. This datum is assumed to be stable. In the Netherlands, the elevation of the levelling points is usually given with respect to NAP (Dutch Ordnance Datum, ~ mean sea level). The benchmarks are generally founded within the uppermost sandy deposits of Pleistocene age. Consequently, the measurements are not influenced by the movement of the Holocene soft coastal soil. Therefore, the levelling data can be used to verify the effect of subsidence due to gas extraction, without the interference of subsidence processes of the soft soil. The assumption is made that there is no significant contribution from other subsidence sources from causes within the Pleistocene deposits or below. Following Verberne et al. (2025a), the expected contribution of compaction by overburden weight of pre-Holocene unconsolidated strata is in the order of 0.5 mm/year in the area, taking into account the thicknesses of these deposits (TNO-GDN 2024). Subsidence derived from levelling data differs from subsidence derived from InSAR data, as InSAR data is influenced by the Holocene soft coastal soil. The levelling data is used as a separate verification of the deep subsidence model.

**Fig. 5:**
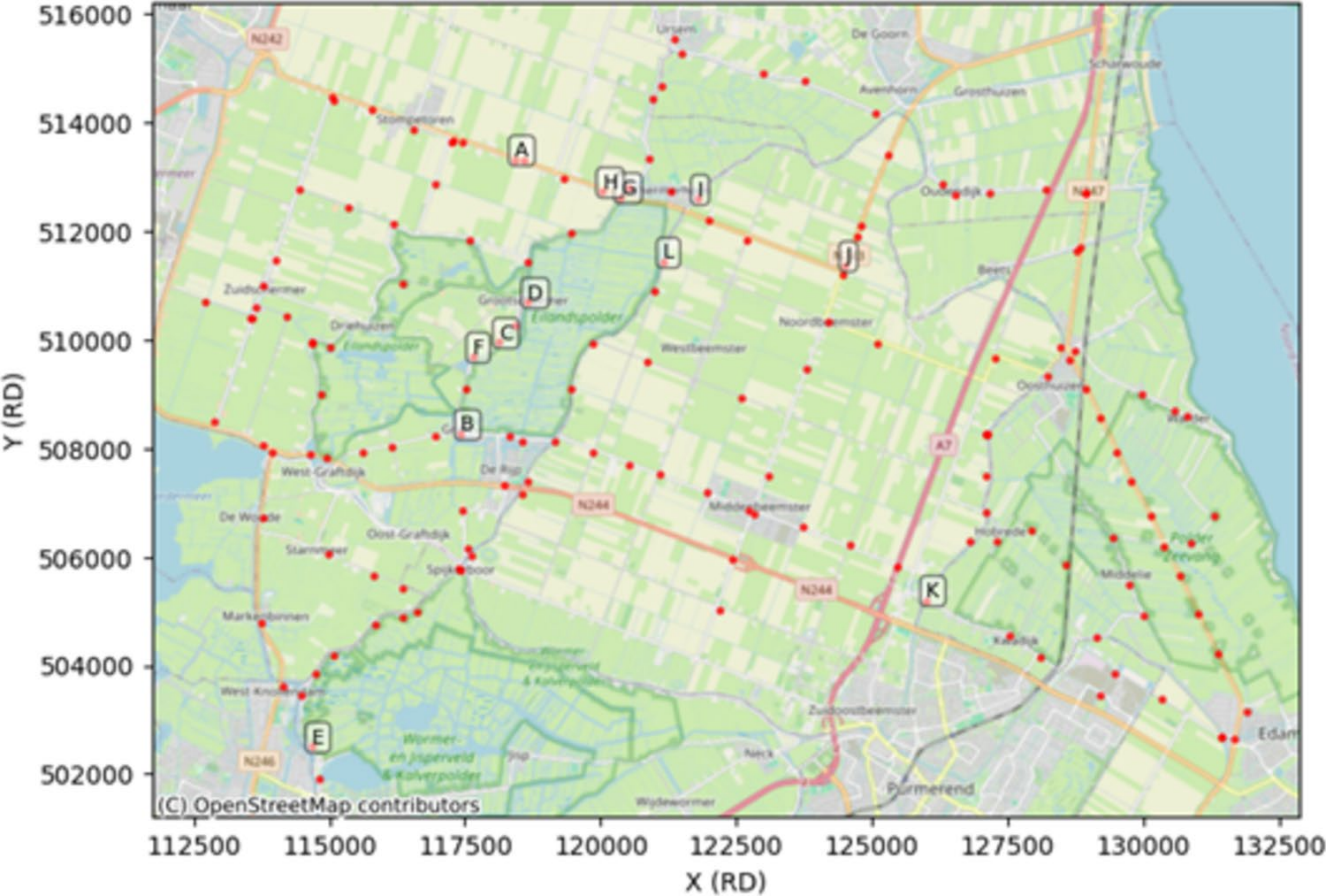
Red dots show the locations of the levelling data used for this study. The letters indicate the location for which the modelled subsidence is plotted against the levelling data in Appendix B. The coordinate system is Rijksdriehoek (RD)-new

The levelling data of the research area were retrieved from Rijkswaterstaat – Ministry of Infrastructure and Water Management (2023). Levelling data is always in reference to a specified stable benchmark. For this study, we have considered all available data in reference to the in 2005 reviewed NAP benchmark. The time period of the datapoints within this dataset is between 1990 and 2020. Locations with less than 3 measurements over time are excluded. Figure 5 shows all the selected levelling benchmark points.

### Subsurface Data

#### 3D Geological Model

GeoTOP is a 3D geological subsurface model developed by the Geological Survey of the Netherlands, with a resolution of 100 × 100 × 0.5 m that schematizes the Dutch onshore subsurface to a maximum depth of 50 m with respect to NAP (TNO-GDN 2024). For each cell in the model, the most probable lithostratigraphic unit (cf. Figure 2), and the probability estimate of the litholoclass is given (Stafleu et al. 2011, 2021). The model has been constructed based on ca. 580.000 boreholes.

**Fig. 6:**
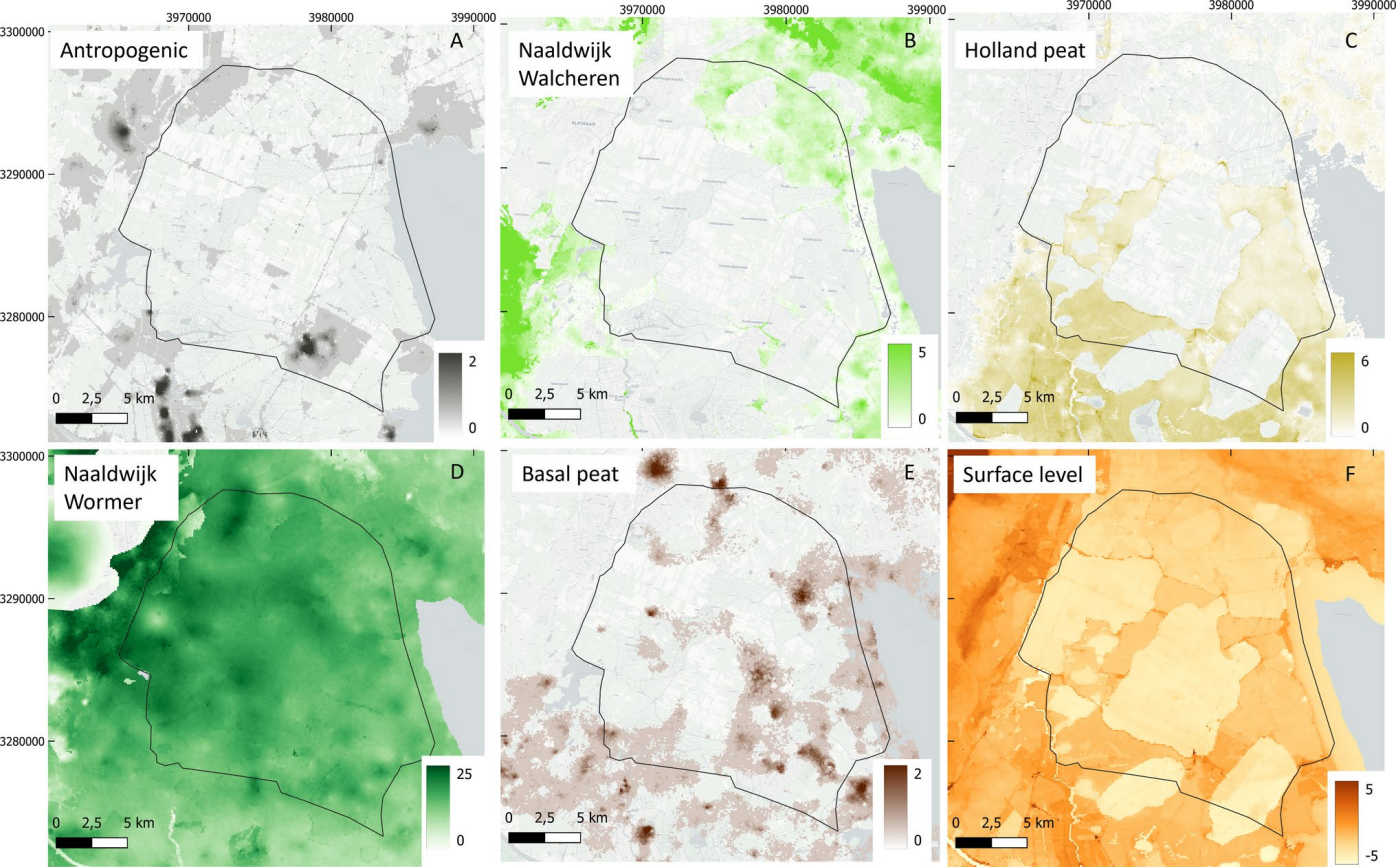
**A**,** B**,** C**,** D**,** E**Thickness of the different geological units present in the study area, as derived from TNO-GDN (2024).** F** Surface level with respect to NAP as derived from TNO-GDN (2024). The coordinate system is Rijksdriehoek (RD)-new

The following lithostratigraphic units of Holocene age are present within the study area: Basisveen Bed, Wormer Member including its Velsen Bed and separately modeled sandy-channels, Hollandveen Member, Walcheren Member, and Anthropogenic brought-up soil (Fig. 6). The lithostratigraphic units are simplified by incorporating the Basisveen Bed into the Wormer Member, because this unit is buried deep (> 10 m), is relatively thin (0.5 m), and is expected to have low compaction potential. This reduces the number of parameters to optimize for the behavior of the shallow soil.

**Fig. 7:**
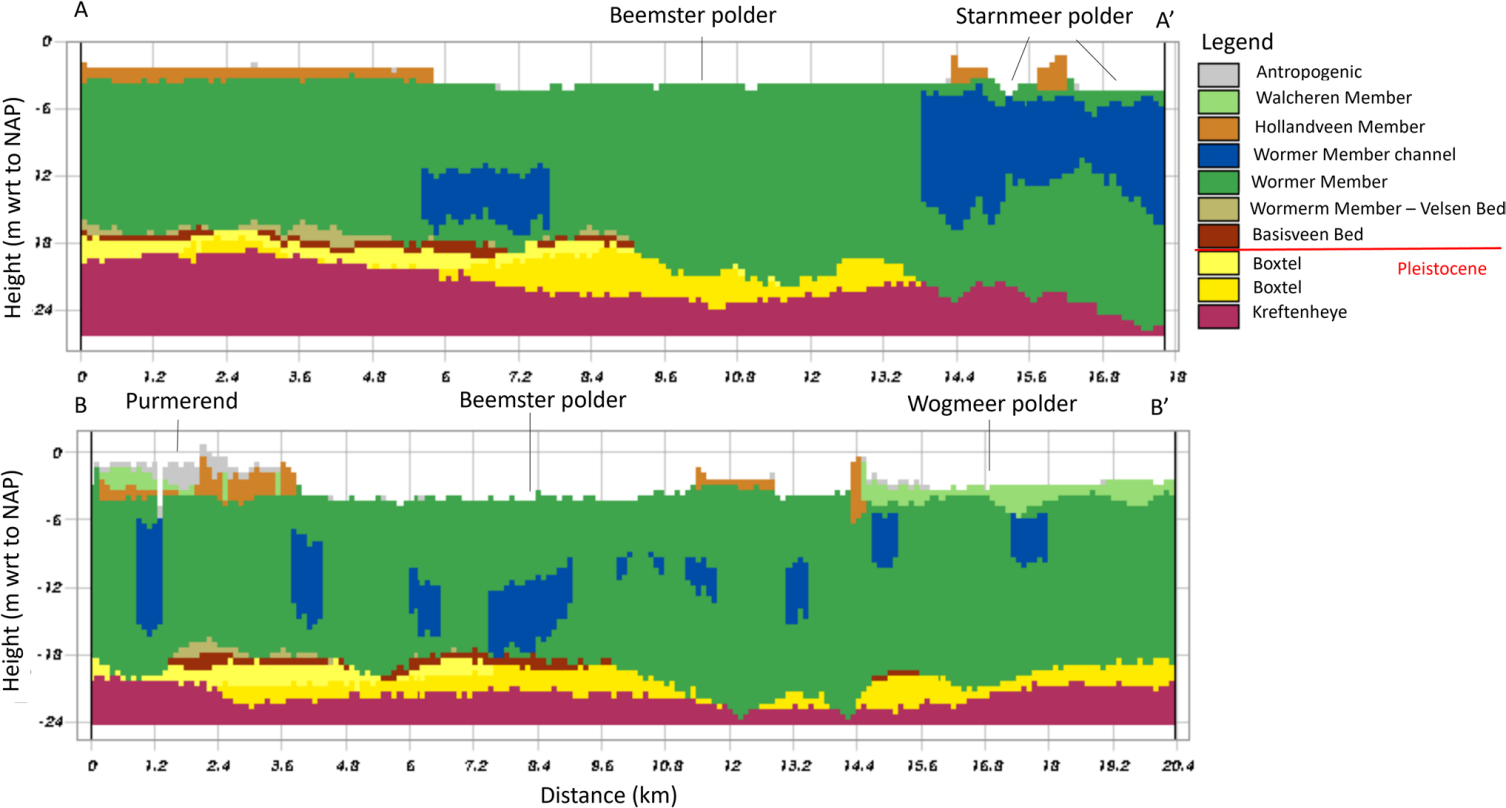
Lithostratigraphic profiles for the study area.** A**-**A**’ plots the east–west profile and** B**-**B**’ the north–south. The profile locations are indicated in Fig. 1D. The stratigraphic profiles are adapted from TNO-GDN (2024)

Figure 7plots two GeoTOP lithostratigraphic profiles for the Holocene sequence of the research area. The city of Purmerend and the polders that are crossed have been indicated on these profiles.

#### Gas Extraction Data

**Fig. 8:**
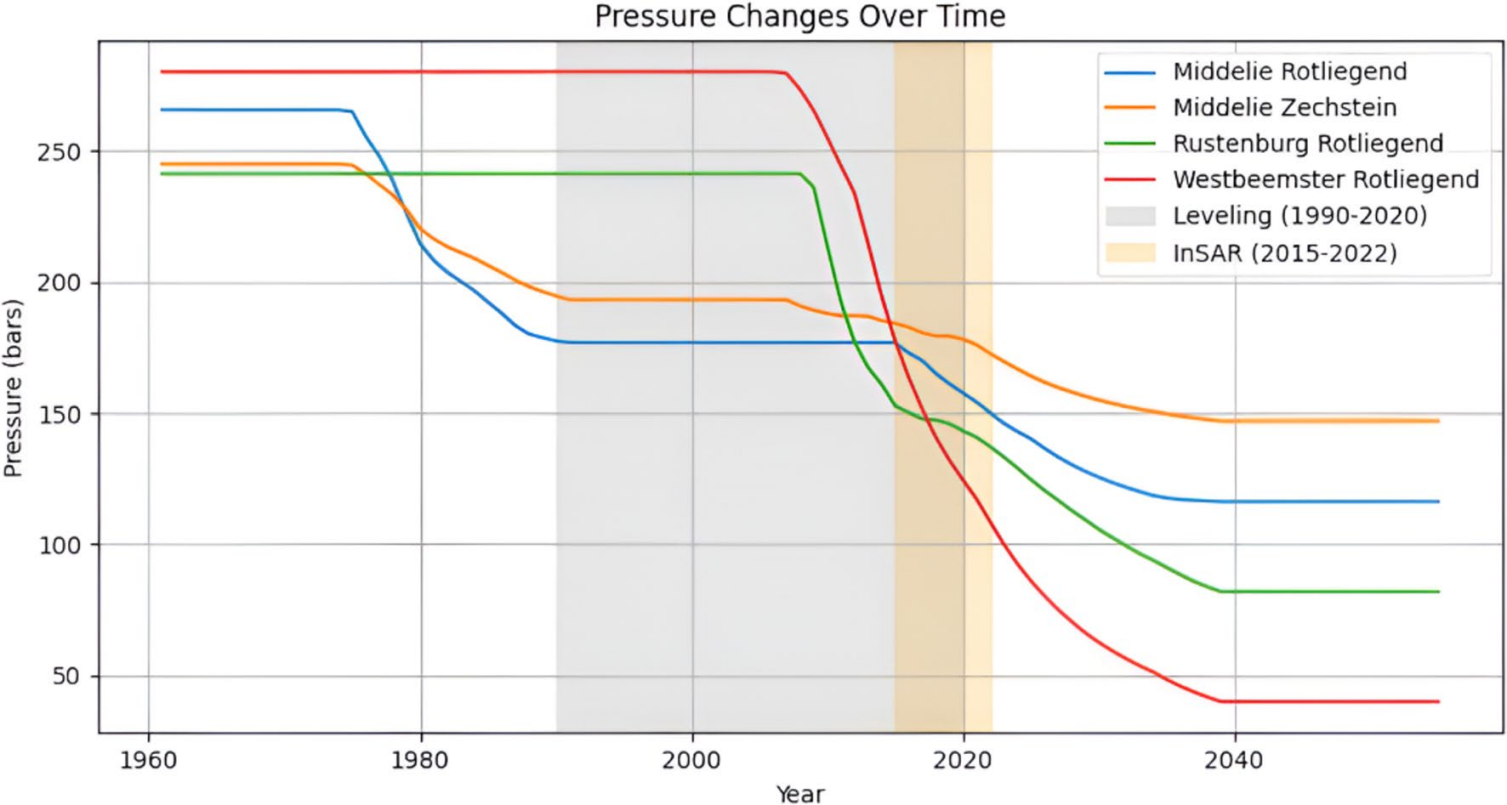
Past and predicted pressure difference resulting from gas extraction for four wells of the four different gas fields. The periods for which levelling and InSAR data is available are also indicated

Data for modelling subsidence as the result of gas extraction have been retrieved from NLOG (2024), an online portal for hydrocarbon data operated by the Geological Survey of the Netherlands, and from a report by NAM (2023). The past and predicted pressures for the production wells of each of the four producing gas fields (i.e. Middelie Rotliegend, Middelie Zechstein, Rustenburg Rotliegend and Westbeemster Rotliegend) are represented in Fig. 8. The figure also indicates the periods in which surface measurements are available. The necessary input parameters for each of the gas fields are given in Table 2. A spatially uniform pressure has been assumed for the gas reservoirs. This was motivated by the fact that the fields are produced through substantial pressure depletion: the associated average pressure decrease is much larger than the pressure variations across the field.

**Table 2:** Necessary input data for the modelling of subsidence by gas extraction, retrieved from NAM (2023)

Property/parameter	Middelie Rotliegend	Rustenburg Rotliegend	Westbeemster Rotliegend	Middelie Zechstein
Depth field (m)	2360	2230	2500	2050
Average depleting thickness (m)	190	170	180	15
Poisson ratio (-)	0.2	0.2	0.2	0.2
Cmref (10^–5^ bar^−1^)	0.89	0.83	0.73	1.28
Cmd (10^–5^ bar^−1^)	0.42	0.39	0.35	0.28
B (-)	0.021	0.021	0.021	0.046
Uncertainty	25%	25%	25%	25%

### Models

Two different models are required to determine the total subsidence effect: one for subsidence by shallow causes, estimating the contribution of all shallow processes comprising compaction, shrinkage and oxidation, and one for subsidence by deep causes, estimating the contribution to subsidence by gas extraction.

#### Shallow Subsidence Model

Thickness reduction of Holocene soft soils in the coastal plains of the Netherlands arises from various processes. Clay and peat are particularly susceptible to compaction, which results from increased effective stress caused by a lowering of the phreatic groundwater level, the weight of overburden, or additional loads of anthropogenic brought-up soil (Schothorst 1977; Koster et al. 2018). Additionally, shrinkage of clay and oxidation of organic material occurs in the unsaturated zone, especially during periods of prolonged aeration (e.g. De Glopper 1969; Barciela-Rial et al. 2020; Blondeau et al. 2024). The modeling of these processes is inherently complex, involving multiple parameters and requiring detailed knowledge of subsurface properties (e.g., Bjerrum 1967; Den Haan et al. 1996; Verberne et al. 2023). The total subsidence within a soft soil layer is often driven by a combination of factors (Fokker et al. 2019; Verberne et al. 2023).

Given that the period considered here (2015–2022) is short with respect to the time passed since reclamation (more than 350 years) and loading has been relatively constant (e.g., stable groundwater levels, no major land-use change) we assume temporal linearity in subsidence rates. This simplification facilitates the detection of spatial variability, which is expected to dominate over temporal variability in this context.

We further assume that the subsidence rate is primarily a function of the stratigraphic unit. This implies that areas with similar lithologies and land-use histories are expected to exhibit similar compression rates. Consequently the following model is adopted:

1 $$ S(\Delta t) = - B_{s} *\Delta t*\delta h $$

where B_s_ is the soil compression parameter, specific for each stratigraphic unit, Δt the time passed and δh the thickness of the stratigraphic unit. This simplified approach offers a practical way to estimate subsidence within the constraints of current data and modeling capabilities.

#### Gas Production-Induced Subsidence Model

No previous parameter optimization had been conducted for the gas fields in the area. We therefore follow the approach of studies in similar gas fields. Here, reservoir compaction by gas extraction is modelled with a rate type compaction model (de Waal 1986; Pruiksma et al. 2015; Van Eijs and van der Wal 2017; Candela et al. 2022). In Appendix A, the rate type compaction model is outlined.

To translate reservoir compaction to subsidence at the surface, many studies follow the nucleus of strain concept of Geertsma (1973). In this model, the subsurface is assumed to be a homogeneous, isotropic, linearly elastic half-space. However, the subsurface is not homogeneous; the subsurface consists of weak and strong layers. As a result, subsidence models using Geertsma (1973) often show a wider and shallower subsidence bowl than realistic (e.g. Van Thienen-Visser et al. 2015). To better represent the non-homogeneous subsurface, and therefore, model a more realistic subsidence bowl, a vertical Double Force strain nucleus (Mindlin and Cheng 1950) can be implemented. The shape of the Double Force influence function is always narrower and steeper than the Geertsma solution. Other solutions include the use of a rigid basement (Van Opstal 1974), a semi-analytical approach with layers of different properties (Fokker and Orlic 2006; Mehrabian and Abousleiman 2015; Park et al 2021a, 2021b; Yan et al 2023), or the use of transverse isotropic elastic parameters (Janna et al. 2012). All these solutions create a narrower and steeper subsidence bowl. We deploy the influence function originating from a vertical Double Force because it is a simple alteration of the Geertsma (1973) concept, it does not need additional input parameters such as the depth of the rigid basement (e.g. Van Opstal 1974), and it is based on physics arguments for depleting layers which are weak in comparison with the surroundings (Fokker and Osinga, 2018).

The idea of calculating subsidence at the surface with the use of an influence function is that the compaction of the reservoir is represented by many nuclei of strain and that the effect at the surface is the superposition of their effects, as with a Green’s function. We define the source depth c, the lateral position with respect to the nucleus location $\left(x,y\right)$, and $R=\sqrt{{x}^{2}+{y}^{2}+{c}^{2}}$. The influence function for a vertical Double-Force source that represents 1 m3 of compaction in the reservoir then is given by (Mindlin and Cheng 1950) with $\nu$ as the Poisson ratio:

2 $$ \left( {\begin{array}{*{20}c} {u_{1} } \\ {u_{2} } \\ {u_{3} } \\ \end{array} } \right)_{DF} = \frac{1 - \nu }{{\pi \left( {2 - 4\nu } \right)}} \cdot \left\{ {\frac{2\nu }{{R^{3} }} - \frac{{3c^{2} }}{{R^{5} }}} \right\}\left( {\begin{array}{*{20}c} x \\ y \\ c \\ \end{array} } \right) $$

Appendix B presents plots showing surface movements from leveling data over time at several locations in the research area, alongside subsidence estimates based on both the NAM (2023) scenario and a maximum scenario (25% larger values for C_md_ and C_mref_). This comparison demonstrates that varying input parameters has minimal impact on the modeled subsidence, especially in light of the uncertainty associated with leveling measurements. Therefore, to minimize the effect of non-unique solutions for parameter values, only the C_m_ parameter is optimized in this study.

### Data Assimilation

Ensemble Smoother with Multiple Data Assimilation (ES-MDA) (Emerick and Reynolds 2013; Evensen et al. 2022) leverages an ensemble of subsidence model outcomes, generated through Monte Carlo-based sampling of model parameters. The initial ensemble is created using the mean and standard deviation of the parameter values (the error); subsidence estimates are calculated for each ensemble member, using the forward models. Discrepancies between modeled subsidence estimates and observed data are then calculated and used to update the ensemble. In this case study, no additional data is made available over time. The optimization of each step will include all available processed InSAR data. Parameter estimates are refined iteratively over multiple assimilation steps, enhancing model accuracy. The application of this methodology is described in more detail in Appendix C and in Verberne et al. (2023, 2024). Appendix D provides the code for the ES-MDA procedure. Appendix C also describes how the performance of the model is assessed. The uncertainty of the model outcome for both the Purmerend subset and the complete research area are quantified with an absolute error (AE) and absolute ensemble spread (AES) (Franssen and Kinzelbach, 2008). These values respectively indicate the difference between the model output and the data and the difference from the ensemble estimates from the average result, representing accuracy and precision. Additionally, to understand the uncertainty of the results spatially, the difference between the modeled results and the data is plotted on maps. Lastly, uncertainty for the complete research area was assessed from the final ES-MDA ensemble using spread and distribution metrics for total, deep, and shallow subsidence contributions. These were compared to the variability in InSAR-derived subsidence rates to evaluate consistency and the impact of observational outliers in the InSAR data.

As shown in the workflow in Fig. 3, the confrontation of modeled subsidence with subsidence data in the present study involves two main steps. First, the subsidence from shallow causes is assessed for a region outside the influence zone of gas extraction (Fig. 4B). The parameters for the shallow processes are optimized with ES-MDA. The subarea location includes the city of Purmerend and its surroundings, and spans both the reclaimed areas and the polders with remaining peat deposits. This enables a clear distinction between the two primary characteristics of the research area, although the final parameter fit may not fully represent the average behavior across the entire study region. The parameter values for the sub-area serve as input for the entire region in the second ES-MDA step. They are adjusted towards an optimal fit for the entire study region (Fig. 4A), in combination with the parameter values for the deep subsidence processes. Table 3 provides the specifics for both calibration steps, including the number of assimilation steps, the standard deviation of the error, and the used inflation factor. Table 4 and 6 provide the prior estimates of the parameters to be optimized. For the shallow model these are the parameter values for B_s_ dependent on the stratigraphical class (Eq. 1), $\delta h$ is determined based on the GeoTOP model per location. For the gas extraction model the parameter to be optimized are the C_m_ parameters for the different gas fields. Table 2 gives the state variables for the gas extraction model.

**Table 3:** Study specific settings for each analysis step

Analysis step	Assimilation steps	Ensembles members	Data error (stdv)	Number of parameters	Inflation factor
Purmerend shallow subarea	4	400	5 mm	4	1.5
Total research area	4	400	5 mm	8	1.7

The data error is given based on a randomized values picked from a normal distribution, with a standard deviation around 0

**Table 4:** Prior and posterior values for the parameter estimates of the Purmerend subarea in mm per meter thickness per year

Parameter	Prior(mm/m/year)	Posterior (mm/m/year)
Anthropogenic	0.10 ± 0.05	0.040 ± 0.003
Hollandveen Mb	0.39 ± 0.05	0.330 ± 0.005
Walcheren Mb	0.14 ± 0.05	0.050 ± 0.003
Wormer Mb	0.10 ± 0.05	0.110 ± 0.008

The AE is 15% and the AES 87%. The values are all mm/year subsidence per meter thickness of a unit

For mitigation measures it is important to identify areas most prone to subsidence, and what the drivers of subsidence are. Therefore, the cumulative subsidence has been modelled until the year 2050. For the gas-related subsidence, the gas extraction values scheduled by NAM have been used (Fig. 8); for the shallow processes, the optimized linear rate has been extrapolated. Based on the optimized parameters from Table 6, we calculate the maximum predicted subsidence, following the gas extraction rates. It should be noted that it is likely that shallow subsidence rates reduce over time, which has not been taken into account. As a result, the absolute values of expected subsidence may be overestimated. We therefore refer to the expected subsidence as the total maximum subsidence. Additionally, the modelling is based on the available InSAR data, which is generally data on top of structures. These future estimates therefore are representative for the built-up environment, not so much for the bare surfaces. Still, the qualitative pattern remains, as a consequence of the shallow stratigraphy and the gas extraction subsidence bowl.

## Results

The results are divided into three parts. First the results on the Purmerend subarea are presented, outside the influence range of subsidence due to gas extraction. Second, the results of the analysis of the total research area are presented, with both shallow and deep caused processes. Lastly, based on the outcome of the analyses, subsidence forecasts are provided.

### Purmerend Subarea, Shallow Caused Subsidence

**Fig. 9:**
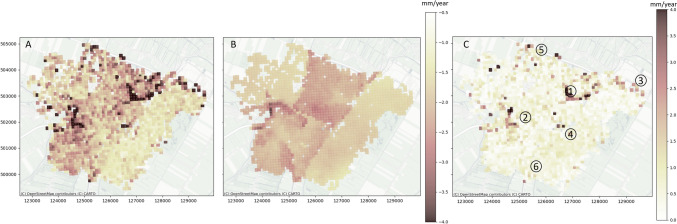
**A** InSAR-derived subsidence estimates in mm/year for the Purmerend subarea, same scale as middle plot.** B **Modelled subsidence in mm/year for the Purmerend subarea.** C** Absolute difference between the InSAR-derived subsidence estimates and the modelled subsidence. The numbers indicate the locations with the largest difference, which will be discussed in the discussion section

Figures 9A and B show the data and the fitted model estimates in the Purmerend area. Figure 9C plots the absolute difference between the data and the subsidence estimates. The estimates generally agree well with the data, except for a number of locations, which are indicated in the figure and identified in the discussion.

The numerical results for the parameter values are summarized in Table 4, with the model performance in the Table description, and show that the peaty Hollandveen Member is the most compaction-prone lithostratigraphic unit. Consequently, most subsidence is observed at the locations where the surficial peat beds of the Hollandveen Member are still present (Fig. 6). The standard deviation of the final fit is relatively small (Table 4); therefore, the posterior parameter values are used as the prior estimated parameters for the shallow model of the total research area in the next step, but with increased uncertainty.

### Complete Research Area, Deep and Shallow Caused Subsidence

Table 5 provides the outcome of the analysis of the complete area with the InSAR-derived estimates in terms of the contribution to subsidence, and Table 6 gives the optimized parameter values for the deep and shallow model and the performance of the model is described in the table description. The mean rates of the total modelled and InSAR-derived estimates agree. However, the InSAR-derived estimates show a higher standard deviation. This is related to the more extreme values for the minimal and maximal subsidence. We therefore also considered the InSAR-derived estimates with the outliers removed. This removal was done using the Interquartile Range (IQR) (Wan 2014) with a threshold of 1.5, which removed 121 of the 2131 locations.

**Table 5:** Statistics of the calculations of the contributions of the subsidence rates of the entire research area

	Total modelled	Deep modelled	Shallow modelled	InSAR-derived	InSAR-derived without outliers
Mean rate (mm/year)	-2.42	-0.24	-2.18	-2.34	-2.17
Standard deviation (mm)	0.60	0.52	0.36	1.34	0.89
Max rate (mm/year)	-5.26	-2.65	-3.95	-21.12	-6.83
50% median (mm/year)	-2.30	-0.01	-2.12	-2.06	-2.00
Min rate (mm/year)	-1.28	0.01	-1.28	13.45	-0.08

AE is 3% and AES is 75%. 121 out of 2131 datapoints are removed from the outlier statistics

Note the large InSAR-derived value for the minimal subsidence rate (positive = uplift), which is not the case anymore when the outliers are not included

**Table 6:** Parameter values for the prior of the total area and optimized for the total area

Shallow parameters (mm /m/year)	Prior	Posterior
Anthropogenic	0.040 ± 0.008	0.001 ± 0.000
Holland peat Mb	0.330 ± 0.068	0.340 ± 0.017
Walcheren Mb	0.050 ± 0.01	0.170 ± 0.010
Wormer Mb	0.110 ± 0.022	0.120 ± 0.006
Deep parameters (10^–5^ bar^−1^)		
Middelie Zechstein Cm	0.270 ± 0.027	0.950 ± 0.092
Middelie Rotliegend Cm	0.420 ± 0.042	1.480 ± 0.036
Westbeemster Rotliegend Cm	0.340 ± 0.34	0.240 ± 0.010
Rustenburg Rotliegend Cm	0.390 ± 0.39	0.410 ± 0.040

The input parameters are derived from the analysis of the Purmerend area and the values from the NAM (2023) report

**Fig. 10:**
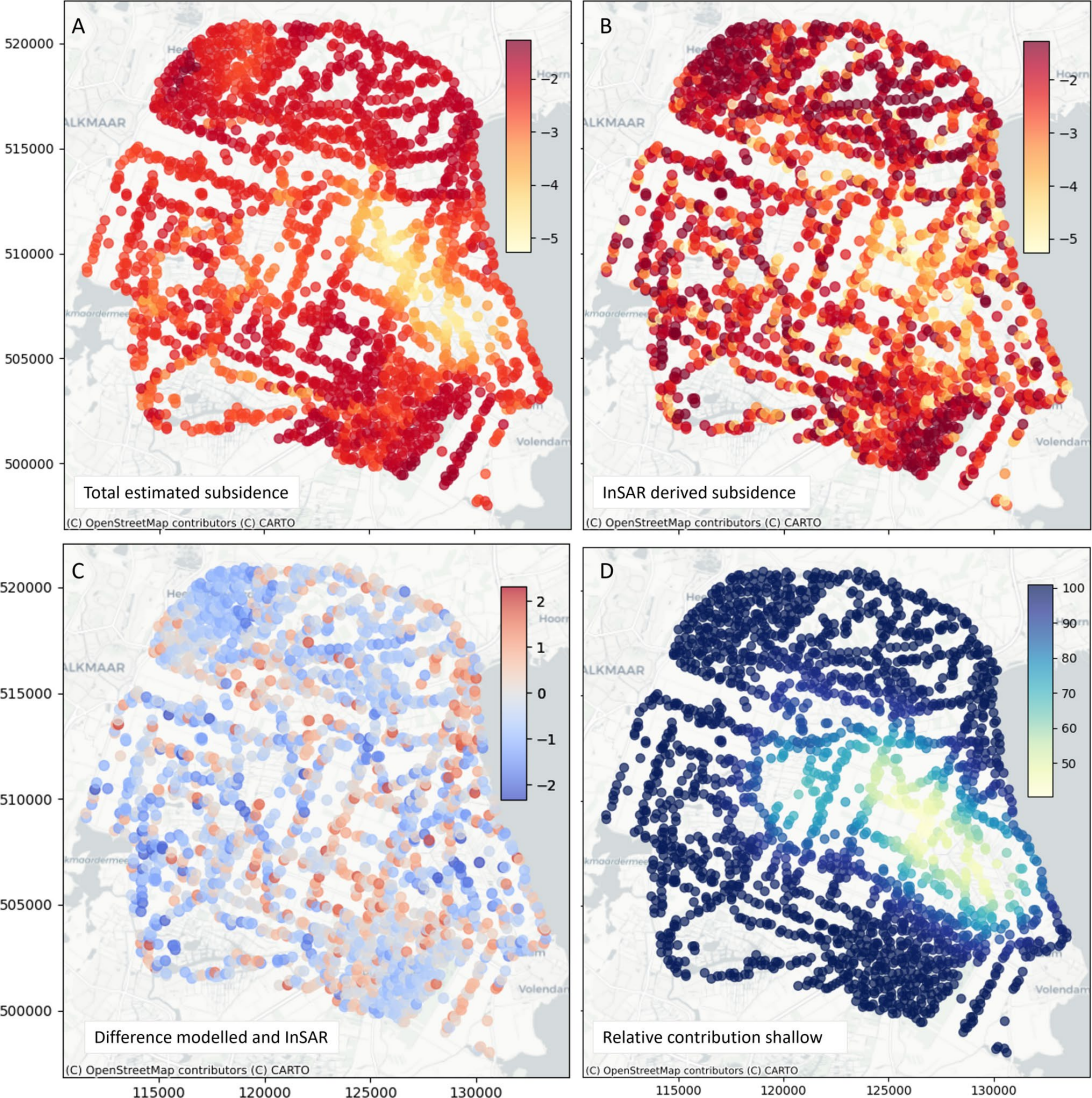
**A** total modelled subsidence in mm per year for the research area.** B** InSAR-derived estimates of subsidence in mm/year.** V** difference between the modelled and InSAR-derived subsidence estimates.** D** relative contribution of shallow subsidence to the total subsidence

Figure 10 shows the modelled subsidence patterns, the InSAR-derived subsidence patterns and the difference between those two, as well as the relative contribution of shallow subsidence with the outliers removed. Figure 10A and B show that the modelled and InSAR-derived patterns of subsidence are similar, but the total rates of the InSAR-derived estimates are more extreme. For example, in the city of Purmerend in the southeast of the study area, both InSAR-derived and modelled subsidence show that there is a spatial difference in the subsidence rates, similar as in Fig. 9, but the values of the InSAR-derived estimates vary more than the modelled rates.

**Fig. 11:**
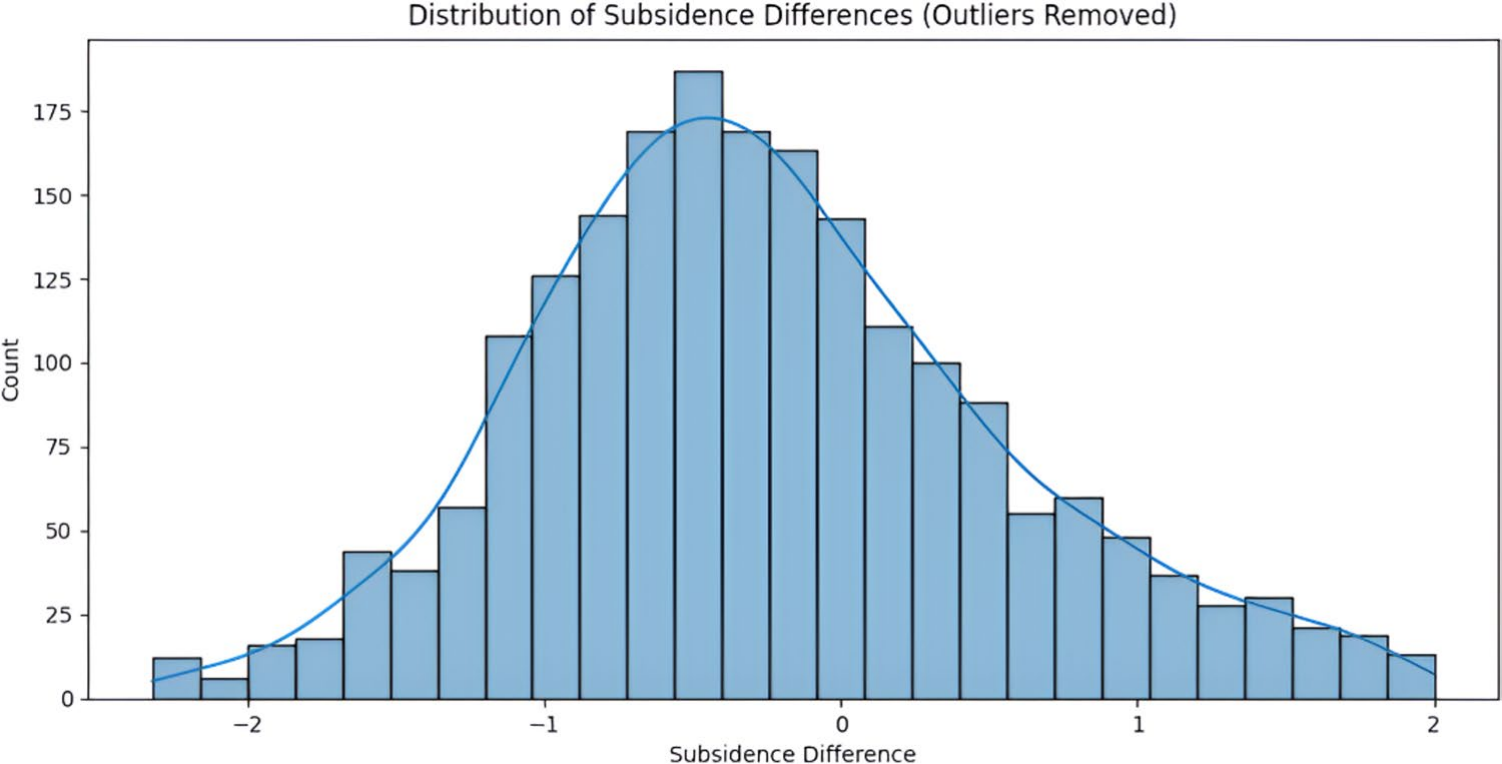
Distribution of the subsidence differences between the InSAR-derived estimates and the modelled subsidence.The Bars represent the number of locations with a certain subsidence difference. The blue line gives the smoothened version of the bar plot

Figure 10C shows the spatial difference between the InSAR-derived estimates and the modelled subsidence. There are some clusters of points that show a similar difference from the InSAR-derived values compared to the modelled subsidence. These clusters can imply consistent local behavior that deviates from the modelled subsidence. Figure 11 shows the distribution of the differences. A normal distribution around zero implies an unbiased model. However, we find the mean to be around -0.4 mm/year, implying a slight bias to underestimate the subsidence in the research area.

**Fig. 12:**
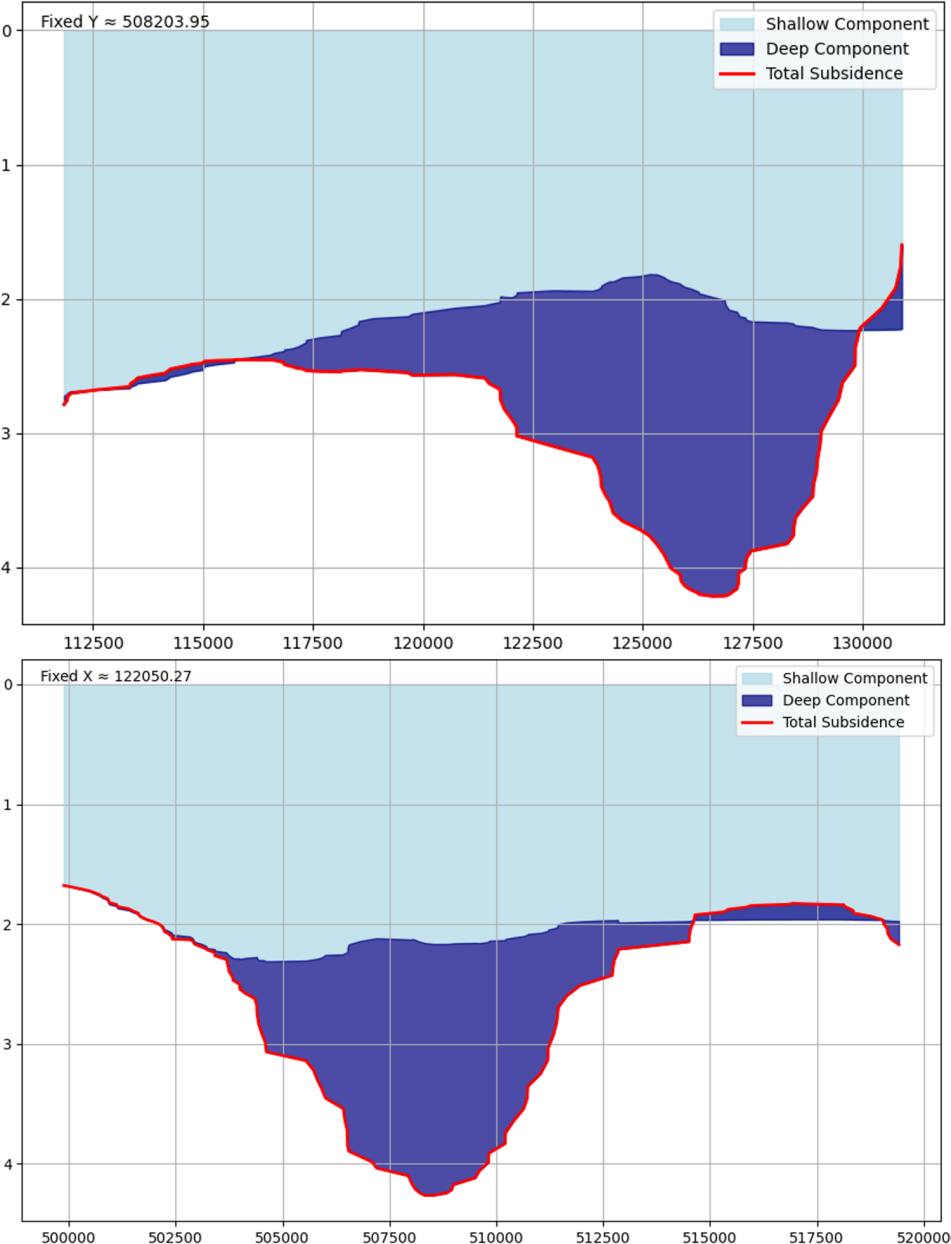
Subsidence contribution deep and shallow along X-axis (top) and Y-axis (bottom) at a chosen coordinate (cf Fig. 1), with a 500 m buffer around the coordinate. Y axis in mm per year

Table 5 shows the ensemble statistics from the final ES-MDA iteration and quantifies model uncertainty across the study area. Standard deviations indicate higher uncertainty for the deep component than for the shallow component. The modelled range is narrower than that of the InSAR data, which shows extreme values (e.g., uplift of + 13.45 mm/year) largely removed after outlier filtering. The reduction in InSAR standard deviation from 1.34 to 0.89 mm/year confirms the strong influence of a small number of outliers in the InSAR data (121 of 2131 points). Table 5 also indicates the mean rates of the deep and shallow contributions to the modelled subsidence. On average, the shallow contribution is much larger. This is also expressed in Fig. 10D where the relative contribution of the shallow processes is plotted. The shallow processes are dominant in the largest part of the research area, but deep processes contribute substantially in the mid-eastern part of the area. This is also visualized in the two profiles along the X and Y-axis (Fig. 12), with all modelled points that fall within 500 m from the chosen axis, as given in Fig. 1. The largest subsidence rates are reached where both deep and shallow contribute to the total subsidence. Note that sometimes the deep subsidence contribution is below the total subsidence (the red line) in Fig. 12. This is because the influence function of a Double-Force exhibits some uplift further away from the center (see e.g. Fokker and Orlic 2006; Fokker and Osinga, 2018).

### Subsidence Forecasts

**Fig. 13:**
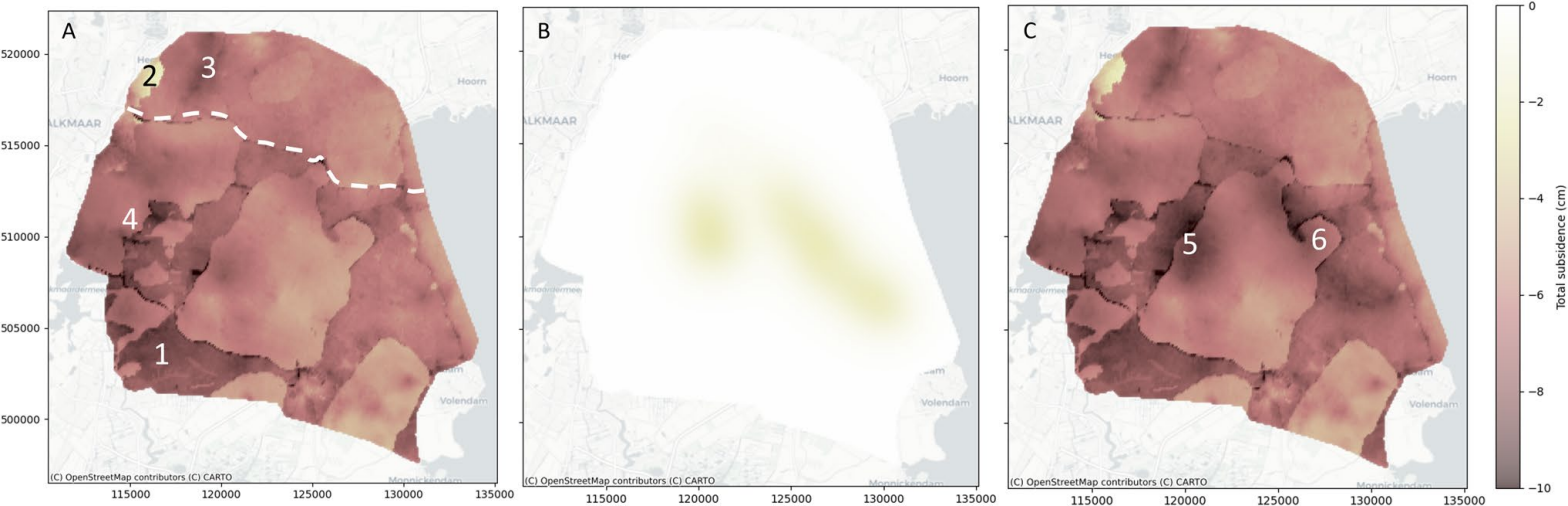
Maximum estimated future subsidence: total subsidence in 2050 with respect to 2020, for shallow (**A**), deep (**B**), and total subsidence (**C**)

Figure 13 shows the maximum total expected subsidence in January 2050 with respect to January 2020. We refer to the expected subsidence as the maximum expected subsidence, because shallow soft soil compaction is likely not to continue linearly on a timescale of several decades but rather progresses log-linearly (e.g. Koster et al. 2018). Additionally, we acknowledge that the uncertainties of the optimization, as expressed in Fig. 10C, allow for limited interpretation of the absolute estimated subsidence values. Yet, the 2050 estimate indicates the maximum expected values and the spatial patterns in the area, which is useful for localizing areas of importance for effective mitigation measures. The absolute values in these maps should be used with care.

Figure 13A provides the estimated shallow compaction in the year 2050 with respect to 2020. Subsidence reaches a maximum of about 10 cm in 30 years. The northern part of the research area (above the white dotted line in Fig. 13A) has relatively low subsidence rates. This region contains the least amount of shallow peat beds and is in general more clay dominated as a result of sedimentation from the northern paleochannel.

For the southern part of the research area (below the white dotted line in Fig. 13A), the subsidence rates can be compared to the surface level (Fig. 6A): where the surface level is the lowest (the reclaimed areas) the subsidence rates are the lowest. This is explained by the presence of peat beds in the higher elevated regions. The regions with the highest elevation are the regions in which no peat mining took place and peat layers are still present. The thickest peat layers are found in the southwest of the study area (location 1 in Fig. 13A), in agreement with increased subsidence rates.

The lowest subsidence rates are found in the northwest (location 2 in Fig. 13A). This part of the region contains tens of meters thick sand layers that are part of the beach barrier system that extends towards the coast in the west. To the east of this (location 3), there are increased subsidence rates correlating with increased thickness of the Wormer Member deposits (Fig. 6E). In the southern part of the research area, the effects of the thickness of the clayey Wormer Member deposits are also visible. Location 4, for example, shows increased subsidence rates related to the increased thickness of the Wormer Member.

**Fig. 14:**
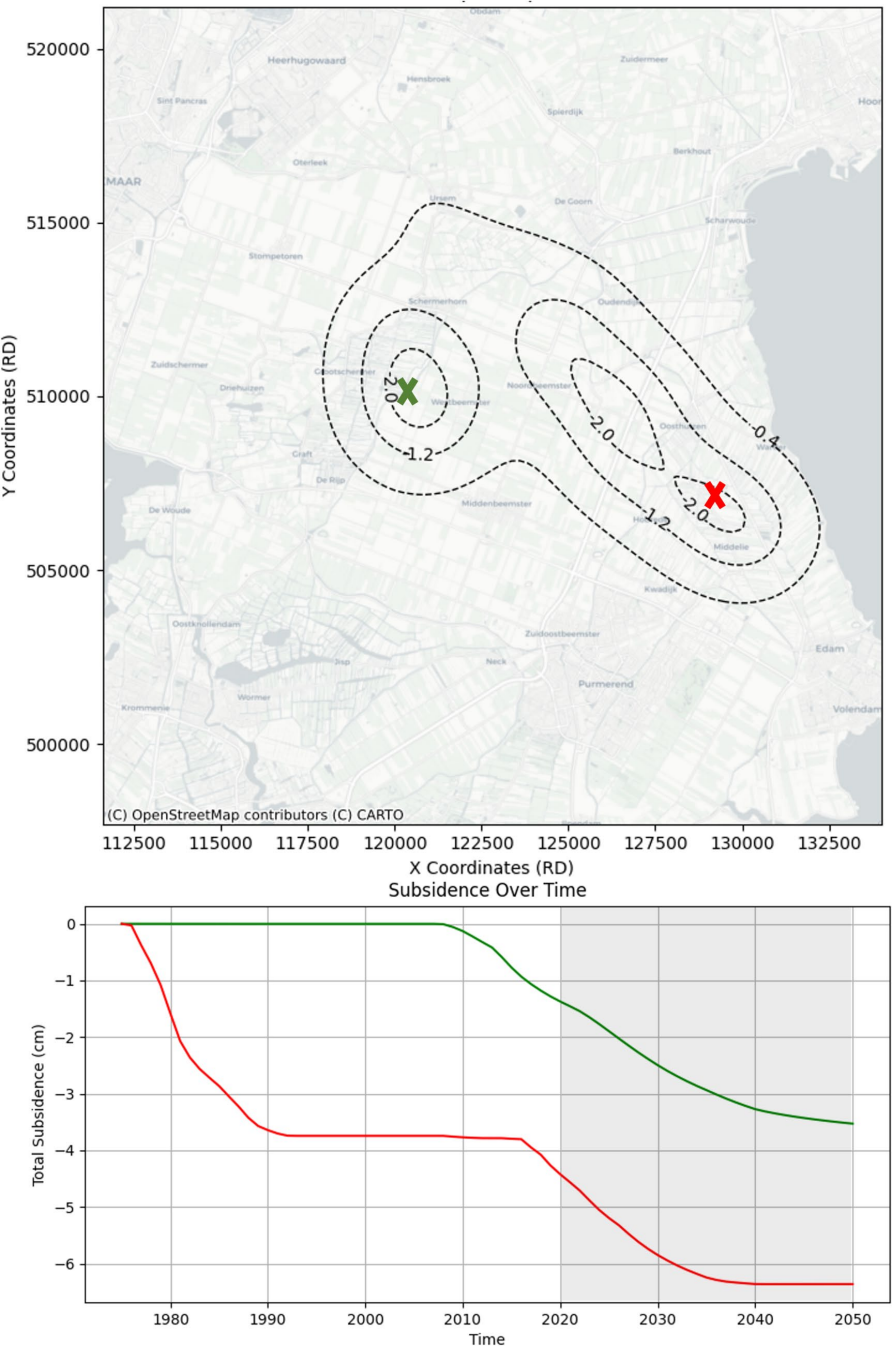
Top: Gas-extraction-related subsidence in the year 2050 since 2020, with a contour plot of 0.4 cm. Bottom: Modelled cumulative subsidence from the start of extraction in 1975 until 2050, for two locations indicated on the top plot. Highlighted is the total subsidence since 2020 until 2050 as depicted in the contour plot

The effect of gas production on subsidence is presented in Fig. 14A, where the spatial extent of the total subsidence in the region caused by the scheduled gas extraction for the period from 2020 to 2050 is mapped. The expected surface movement due to gas production since 1975, for locations above the Middelie Rotliegend field and the Westbeemster field are also given in Fig. 14B. The subsidence due to gas production is non linear with time, because it is controlled by the gas extraction rate. The total maximum expected subsidence rate due to gas extraction is slightly over 6 cm since the start of the extraction in 1975. The total expected gas-related subsidence in the region above the Rustenburg and Middelie gas fields is substantially larger than in the area of the Westbeemster gas field.

Figure 13C shows the combined effect of shallow and deep subsidence. Locations 5 and 6 indicate the two areas where the gas fields have an effect on the total subsidence.

## Discussion

### Subsidence Processes

The present study quantifies the contributions of both shallow and deep causes of subsidence in the coastal plain of the Beemster polder, the Netherlands. While shallow causes dominate subsidence across most of the area, the contribution of deep and shallow processes is roughly equal above the Middelie gasfields. The integrated approach helps to understand the heterogeneity in the subsidence patterns, enabling specified mitigation measures. We discuss the specifics of the deep and shallow processes separately, as well as the implications and challenges of the integrated approach.

#### Subsidence Related to Shallow Processes

Shallow subsidence processes in the study area are caused by a combination of processes, with peat and clay both playing a significant role. The Hollandveen Member, a peat-dominated layer, gives the highest parameter values for subsidence (Table 6), and there is a clear correlation between peat thickness and the magnitude of subsidence (Fig. 14). However, the contribution of clay layers, particularly the Walcheren and Wormer Members, cannot be overlooked. Their total thickness makes their contribution to subsidence substantial. This is in line with earlier studies of Fokker et al. (2019) and Verberne et al. (2023). While processes of subsidence related to peat dominate the soft soil subsidence research in the Netherlands (e.g. Brouns et al. 2015; Koster et al. 2018; Van Asselen et al. 2018), we show here that clay processes are substantial and should also be taken into consideration.

When comparing the shallow parameters of the Purmerend subset to the total research area, one distinct difference emerges (Table 4 and Table 6): the Walcheren Member shows a larger compaction parameter when evaluated with the data in the total research area. This is likely a false confidence in the compaction coefficient of the Walcheren Member in the Purmerend subset, where the deposit is barely present. The parameter optimization of the other stratigraphic classes results in similar compaction coefficients in the two sets (the total area and subset), suggesting that the behavior of these stratigraphic classes is consistent across the study region.

Despite the consistent optimized parameters in the study area for the shallow stratigraphic layers, the obtained shallow subsidence behavior holds a few important limitations. Two main sources for these limitations are the nature of the subsidence observations, derived from InSAR data, and the actual shallow subsidence processes included in our shallow stratigraphy-based subsidence models.

The limitations of InSAR data in the context of shallow subsidence analysis are clear from the analysis of the Purmerend subset. Within the Purmerend subset, discrepancies between modeled and InSAR-derived subsidence rates reveal the influence of localized surface features. Specific locations with high subsidence rates are dominated by objects without a pile foundation. We have identified six locations (numbers 1 – 6 in Fig. 9C). From these numbers, (1) is dominated by sports field, (2) consists of a recently reconstructed road and neighborhood, where preloading activities likely have influenced the rates, (3) are data points in a business park with extensive parking lots, (4) is a cemetery with many tombstones, (5) has additional sports fields, and (6) is a recently constructed road. Grid locations with more objects without a pile foundation likely show subsidence rates above average, because the objects experience more subsidence due to shallow processes. Of course, the averaging of InSAR data over 100 × 100 m grids smooths out localized variations, but the distribution over data points on objects with and without a pile foundation will affect the average. The averaged values are useful for identifying regional patterns and areas of higher subsidence risks in the context of the subsurface properties, but localized studies into individual objects or streets are necessary to determine the actual compaction rates of all the shallow layers. The current estimates are therefore an underestimation of the total rates of unfounded objects, since measurements on top of objects with a pile foundation are included. This means that our results are limited to being an indicator of average shallow subsidence behavior in the region within the built environment on a scale of hundreds of meters.

Since the analysis is representative for the built environment, this has consequences for the further analysis of what processes are represented by the obtained shallow subsidence rates. InSAR reflections predominantly originate from built objects. These objects have an inhibitory effect on oxidation and shrinkage-swelling cycles (e.g. De Lange et al. 2015). Stable groundwater levels in urban areas further diminish the likelihood of shrinkage and oxidation. These findings align with prior research (e.g. Ao et al. 2024; Ciampalini et al. 2019; Koster et al. 2018; Parsons 2021) identifying compaction as a primary cause of subsidence in built environments. While oxidation and shrinkage may still play a role, they are likely secondary to compaction in the context of this study. This does not mean that these processes are not important in the study area; they are, however, not significant within the used dataset of PS-InSAR points.

#### Subsidence Related to Gas Extraction

Optimization of the parameters for compaction of the gas fields was conducted coeval with the optimization of the parameters for shallow compaction in the total research area. No separate optimization was conducted, since the uncertainty in levelling data relative to the potential variability in compaction parameters indicates that further optimization would provide limited improvement (Appendix B), and the InSAR-derived estimates are a combination of deep and shallow processes. The prior estimates of compaction parameters, derived from extensive studies on similar gas fields in the Netherlands, align well with the levelling data and exhibit minimal variability.

The optimized values of the compaction coefficients with respect to the prior values (Table 6) show larger compaction rates for the Middelie gas fields, no change for the Rustenburg field, and slightly smaller values for the Westbeemster field. The larger compaction rates for the Middelie gas fields can point towards deep or shallow causes. An explanation at depth can be found in potential depletion of the aquifers connected to the Middelie Rotliegend gas field (NAM 2023). IF they deplete along with the gas reservoir, a larger volume of rock is compacting and more subsidence results. A shallow cause can be found in an underestimation of shallow compaction above the gas field. The region affected by compaction of the Middelie gasfields includes both the village of Oosthuizen, characterized by pre-1800 buildings with potentially poor foundations, and a neighborhood currently in construction (Kadaster 2024). This area therefore could show subsidence rates above average due to anthropogenic influence of building load. The larger values for the gas reservoir compaction then would compensate for this shallow effect, because it cannot be captured by the global values for the shallow-compaction coefficients for the entire studied region. To determine whether compaction in the Middelie gas fields exceeds initial expectations, or increased building loads in the region are responsible, a local case study is needed.

#### Challenges of Multi-Depth Causes

The ability to distinguish between different causes of subsidence heavily depends on the availability, type, and quality of data. This becomes especially critical when subsidence arises from multiple depth-related processes. In the subregion dominated by shallow subsidence, differences in our modelled subsidence to the InSAR-derived rates can, of course, only be explained by the shallow processes. In the broader research area, where both shallow and deep processes contribute, assessing differences in subsidence patterns becomes more complex. In this study, higher compaction rates related to the gas fields may actually result from two factors: an underestimation of gas-extraction-related subsidence or an influence of shallow foundation depths in the older parts of the village and new neighborhood developments.

Figure 11C demonstrates the dominance of shallow subsidence processes in the area, which may limit the ability to refine parameter estimates for deep subsidence causes concurrently. To improve the disentanglement of shallow and deep processes, additional data are essential. Building-scale data, including foundation depths, would be particularly valuable, but even neighborhood-scale foundation information could provide important insights. Combined with existing InSAR measurements, such data would increase confidence in the parameter estimates for both shallow and deep processes. Especially if for InSAR data a longer monitoring period can be taken into account, so that potential long-term trends that are not visible with the current monitoring period can be accounted for. For deep subsidence processes, regular measurements are crucial to separating shallow and deep contributions. Establishing GPS stations on stable Pleistocene formations would allow precise tracking of deep movements over time. These enhancements in data collection would significantly improve parameter optimization for subsidence processes at all depths, even outside the region of the GPS measurements. This enables more comprehensive analyses and effective mitigation strategies. It would also enable including intermediate depth movement into the analysis, which can have a minor contribution, in the order of about half a mm, to the total subsidence in the study area (Kooi et al. 1998; Verberne et al. 2025a, b). A limitation of this study is that the intermediate depth is ignored, because the expected rates fall within the uncertainty of the measurements. Yet with improved measurements it might be possible to also distinguish the intermediate contribution of subsidence.

### Data Assimilation

The Ensemble Smoother with Multiple Data Assimilation (ES-MDA) (Emerick and Reynolds 2013) method was selected for its efficiency in complex, high-dimensional datasets. However, the technique is sensitive to ensemble collapse, where parameter variability diminishes too strongly during successive updates. The model then does not capture the full range of plausible parameter values. Other challenges are overfitting to observations and spurious correlations between parameters. This is particular to heterogeneous and multi-scale systems like the one studied here (Verberne et al. 2023, 2024; Kim and Vossepoel 2024). InSAR, while valuable for capturing spatiotemporal trends, can exacerbate these issues due to its large data volume.

To mitigate these issues, an empirically determined inflation factor, larger than those reported in other studies (e.g. Miyoshi et al. 2010; Whitaker and Hamill 2002) was applied. This was necessary due to the spatial variability and the diverse subsidence processes acting at different depth levels. This method is effective, but alternative approaches such as adaptive inflation (Raanes et al. 2019), localization (Anderson 2007), and regularization (Evensen 2009) may offer more robust solutions and merit further testing in subsidence contexts.

Improving the data space may also help reduce collapse and spurious correlations. A full covariance matrix (Chen & Oliver 2010), or dimensionality reduction techniques like clustering or Principal Component Analysis might aid in the improvement (Tang et al. 2022). Alternatively, other inversion methods, such as Markov Chain Monte Carlo (MCMC) methods should be considered (e.g. Bierman and Towe 2019; Zhang and Burbey 2016). Verberne (2025) provides an overview of integration methods for subsidence studies in more depth.

### Effects on the Beemster Region

Subsidence has profound impacts on the studied region, where subsidence rates range from less than 2 to over 5 mm per year, with current surface levels between 0 and 5 m below mean sea level in areas of former peat bog mining. Our analysis shows that the dominant driver of subsidence in the area comes from shallow sources. This means that the most effective mitigation measures should focus on shallow causes. Groundwater level management, light building materials, and stable foundation levels for buildings can mitigate the subsidence. It is important to take into account the lithostratigraphy, thus the presence and thickness of subsidence prone peat and clay layers. Additionally, in the part around the Beemster polder that is already subsiding relatively fast due to shallow processes, subsidence due to gas extraction adds to the mix. This increases the stress on effective shallow subsidence mitigation, when extraction continues.

Subsidence in the area has a number of consequences. This includes an increased flood risk and susceptibility to salinization, both compounded by sea-level rise (Den Heijer et al. 2014; Oude Essink 2001). Increased floodings can eventually lead to the drowning of the Beemster UNESCO polder landscape. Momentarily the preservation of the landscape requires additional drainage, which will become a more costly procedure over time with raising water levels. Another consequence is the emission of greenhouse gas related to peat degradation (e.g. Blondeau et al. 2024; Carpentier et al. 2024). Studies utilizing monitoring stations proximal to the study area confirm these impacts (Aben et al. 2024; Buzacott et al. 2024). Although our study does not differentiate between compaction and oxidation of peat layers, peat has been degrading faster than any other soil type in the study area, stressing their importance in the subsidence issue.

Damage to buildings and infrastructure are direct and local short-timescale consequences of subsidence, particularly in peat areas. Yet, clay layers also contribute substantially to subsidence in the region. While flooding risk depends on the magnitude of the subsidence occurring, damage risk depends on differential subsidence, i.e. the variation of surface movement with position, or the extensional or rotational strain (e.g. Nicodemo et al. 2020; Cooper 2008). The subsidence risk for individual buildings depends on factors such as age, construction type, and foundation type. For example, neighborhoods developed from the 1970s onwards are generally constructed on leveled-up sand, which pre-settles underlying soft layers prior to the construction of houses with concrete pile foundations. These buildings have a lower risk of subsidence; however, localized washouts of embankment material occasionally cause sinkholes (e.g. NH Nieuws 2021). Therefore, to create risks maps on subsidence, differential subsidence values combined with buildings characteristics would be a vital next research step for the area.

### Other Regions

In the Netherlands, regions such as Groningen (van Thienen-Visser and Fokker 2017; van Oeveren et al. 2017) and Friesland (Koster et al. 2021; Verberne 2021) are also impacted by both subsidence from processes within soft coastal soil and from hydrocarbon extraction at greater depths. In these regions, full disentanglement of shallow and deep subsidence causes has yet to be achieved. Such disentanglement is important for designing effective mitigation measures. The challenges in optimizing parameters for both deep and shallow subsidence in these areas mirror those observed in the Beemster region of this study. Improving data on building foundations could play a significant role in enhancing total subsidence estimates across all these regions.

Numerous areas around the globe exhibit subsidence from causes at multiple depths. For instance, Bangkok, Jakarta, Mexico City, and Shanghai all experience subsidence due to loading by urbanization and a heterogeneous subsurface superimposed on groundwater extraction at larger depths (Abidin et al. 2011; Cabral-Cano et al. 2008; Phien-Wej et al. 2006; Ye et al. 2016). In the Mississippi Delta in the United States, both groundwater withdrawal and hydrocarbon extraction lead to substantial subsidence. However, the causes have only been considered in separate studies (Day et al. 2020; Jones et al. 2016).

An example of an area with similar challenges as in the study area presented here can be found along the northern Adriatic coast in Italy (Teatini et al. 2005; Antonellini et al. 2019). There, human-induced subsidence is driven by groundwater and hydrocarbon extraction along with surficial soil compaction due to the load of buildings and infrastructure. A similar approach to ours would therefore be beneficial to understand the full impact of subsidence in that area..

### Recommendations for Future Research

The next steps for the study area should be to prioritize targeted data collection for more accurate assessment of subsidence. This enables resolution refinement, damage potential assessment of buildings and improved disentanglement. Key areas for data improvement include expanded groundwater monitoring data, a high-resolution lithostratigraphic analysis on smaller areas to understand heterogeneity, continuous subsidence measurements with a foundation on top of the Pleistocene above the gas fields for precise deep deformation trends, building foundation level data and, perhaps most critical, improved subsidence measurements outside the built environment. The latter can aid in understanding the subsidence impact in natural and farming areas, including potential emissions related to organic matter degradation.

Looking beyond the study area, the future of subsidence research lies in an integrated, interdisciplinary approach. Effective mitigation of subsidence requires a combination of expertise across domains and depths rather than limiting research to isolated fields.

## Conclusions

Land subsidence often stems from multiple processes, therefore an approach that takes into account all the contributing processes in a region is essential to understand and mitigate subsidence effectively. In this study, we have targeted the Beemster polder region in the western coastal zone of the Netherlands, an area where subsidence is the result of multiple human-induced processes, at different depth levels. We have matched subsidence estimates derived from InSAR data to a superposition of model outcomes for subsidence caused by gas extraction and by shallow soft soil processes.

The analysis of the Purmerend subregion, an area solely influenced by shallow subsidence, demonstrated that subsidence patterns are primarily a result of the variation in lithostratigraphy. The peat-dominated Hollandveen Member accounts for the highest subsidence rates, however, thick clay layers also contribute significantly. The resulting average subsidence rates range between 1 and 4 mm/year. The results demonstrate the possibility of identifying regions with the highest subsidence rates to guide targeted mitigation measures.

For the entire study area, simultaneous optimization of gas extraction-related parameters and shallow subsidence revealed a consistent behavior of lithostratigraphic units. The parts of the southern region of the study area where the Hollandveen Member is the thickest show the highest shallow subsidence rates. Gas-extraction-related subsidence is projected to contribute a maximum of 6 cm by 2050, relative to 1975. Shallow processes are dominant, with rates reaching over 5 mm/year. These values are valid for the average behavior in the built environment.

This study demonstrates the complexity of disentangling subsidence processes in areas affected by both shallow and deep drivers. By simultaneously optimizing parameters for multiple causes of subsidence, we provide a framework for integrated modeling approaches that can be applied to other regions with similar challenges. Understanding the contributions of different subsidence mechanisms is critical for local management but also for broader applications in land use planning and sustainable development. Future studies that incorporate finer-scale data, including building-specific measurements and deeper geological information, will improve our capacity to predict and mitigate subsidence impacts and ensure better resilience in subsidence-prone areas worldwide.

## Data Availability

For this study we have used freely available data sources. The InSAR data is downloaded from EGMS (EGMS 2024a), from which we used the ortho product level. Levelling data can be downloaded from Rijkswaterstaat – Ministry of Infrastructure and Water Management (2023). The gas production rates and pressure values to model gas extraction are available through NAM (2023). The outlines of the gas fields were obtained through TNO-GDN (2024). The other necessary input parameters are reported in this manuscript. Appendix D gives the Python code for the ES-MDA procedure in this study. The processed data and Python code for the forward modelling and pre-processing that support the findings of this study are available from the corresponding author upon reasonable request. During the preparation of this work the author(s) used co-pilot in order to improve readability and language. After using this tool/service, the author(s) reviewed and edited the content as needed and take(s) full responsibility for the content of the publication.

## Appendix A: Compaction of a reservoir

The compaction of a reservoir can be calculated assuming poroelasticity. If the lateral extent of a compacting reservoir is much larger than its thickness, the lateral strain can be neglected and reservoir compaction assumed to be uniaxial in the vertical direction. Compaction related to pore pressure depletion can be calculated as (Fjaer et al. 2008):

A1 $$\begin{array}{c}\frac{\Delta h}{h}={C}_{m}\alpha \Delta {p}_{f}\end{array}$$

In this equation $\frac{\Delta h}{h}$ is the change in reservoir thickness, $\alpha$ is the Biot’s poroelastic coefficient, ${C}_{m}$ is the compaction coefficient and $\Delta {p}_{f}$ is the change in pore fluid pressure. It is assumed that compaction has no significant impact on the pressure. The reservoir rocks intrinsic properties relate to ${C}_{m}$ according to:

A2 $$\begin{array}{c}{C}_{m}=\frac{\left(1+\upnu \right)\left(1-2\upnu \right)}{E\left(1-\upnu \right)}\end{array}$$

In which $E$ is the Young modulus and $\nu$ the Poisson ratio.

From the parameters of the equations A1 and A2 the $h$ and the $\alpha$ are generally well constrained. This is also the case in the study area. ${C}_{m}$ has the highest uncertainty and variation (e.g. NAM 2017), and is therefore the value that is optimized through inversion.

The available data consists of pressure data through time. Pressure change is assumed to be equally distributed throughout each respective reservoir. The reservoir is discretized in 100 × 100x $h$ grid blocks, for which the vertical uni-axial compaction is determined with the rate-type compaction model. The model assumes a time-decay between pressure depletion and compaction which fits well field observations from earlier studies (e.g. Hettema et al. 2002; Candela et al. 2022). When loading rate changes, there is a first direct elastic strain response, which is followed by a gradual creep strain. In this study, the rate type isotach compaction model follows an explicit Euler finite-difference scheme with a constant time step ${\Delta t}$ (Pruiksma et al. 2015; Candela et al. 2022).

Per grid block the compaction can be calculated in five steps:

1. The current vertical stress $\sigma \left(t\right)$ and strain $\epsilon (\mathrm{t})$ and the creep strain rate can be calculated as:

A3 $$\dot{{\epsilon }_{s}}=\left(\frac{\epsilon \left(t\right)-{\epsilon }_{0}}{\sigma \left(t\right)}-{C}_{md}\right)\dot{{\sigma }_{r}}{\left(\frac{\epsilon \left(t\right)-{\epsilon }_{0}}{\sigma \left(t\right){C}_{m,ref}}\right)}^{-1/b}$$

In this equation the vertical stress is derived from the mean density ${\rho }_{mean}$ of the overlying layers multiplied by the thickness of the overlying layers, or the depth of the top reservoir, ${z}_{r}$. The effective stress is the difference with the pressure:

A4 $$ \sigma \left( t \right) = \rho_{mean} gz_{r} - P\left( t \right) $$

At the start of the production ($t=0$), the elastic strain ${\epsilon }_{d}\left({t}_{0}\right)$ and the creep strain ${\epsilon }_{s}\left({t}_{0}\right)$

are assumed to be zero. Therefore the total strain is also zero. Then, the reference total strain is determined as:

A5 $${\epsilon }_{0}=-{C}_{m,ref}{\sigma }_{r}$$

In which ${\sigma }_{r}$ is the reference vertical stress, where ${\sigma }_{r}=\sigma \left({t}_{0}\right)$. For these equations there are four parameters that need to be calculated. These are three material parameters ${C}_{m,ref}$, ${C}_{m\mathrm{d}}$ and $b$, and one state parameter $\dot{{\sigma }_{r}}$. ${C}_{m,ref}$ is the reference compaction coefficient and corresponds to the loading rate prior to reservoir depletion. The value is relatively high. ${C}_{m\mathrm{d}}$ is the direct compaction coefficient, it shows the direct effect of change of loading rate. The value is relatively low, in order to model the stiff response of rock to the onset of pressure. $b$ is a laboratory based empirically derived constant. The state parameter $\dot{{\sigma }_{r}}$ is the reference vertical stress at the start of depletion of the reservoir.

2. The second step is to calculate the increase in creep strain:

A6 $$\begin{array}{c}\Delta {\upepsilon }_{s}=\dot{{\upepsilon }_{s}}\left(t\right)\Delta t \#\end{array}$$

From which the creep strain is updated as:

A7 $${\upepsilon }_{s}\left({t}_{+1}\right)={\upepsilon }_{s}\left(t\right)+\Delta {\upepsilon }_{s}$$

3. Then, the time is updated as ${t}_{+1}=t+\Delta t$

4. Assuming a linear stress-strain relationship, the elastic strain can be calculated with:

A8 $${\upepsilon }_{\mathrm{d}}\left(\mathrm{t}+\Delta t\right)={\mathrm{C}}_{\mathrm{md}}\left(\upsigma \left(\mathrm{t}+\Delta t\right)-{\upsigma }_{\mathrm{r}}\right)$$

5. The last step is to calculate the total cumulative strain

A9 $$\upepsilon \left(t+\Delta t\right)={\upepsilon }_{s}\left(t+\Delta t\right)+{\upepsilon }_{d}\left(t+\Delta t\right)$$

From which the total cumulative compaction can be calculated as:

A10 $${V}_{comp}\left(t+\Delta t\right)=-{V}_{0}\upepsilon \left(t+\Delta t\right)$$

These steps can be repeated for each time step.

This leaves us with the four parameters ${C}_{m,ref}$, ${C}_{m\mathrm{d}}$, $b$, and $\dot{{\sigma }_{r}}$. For $\dot{{\sigma }_{r}}$ a value commonly used for studies in similar areas (TNO 2013) is taken For each of the four reservoirs the ${C}_{m,ref}$, ${C}_{m\mathrm{d}}$ and $b$ prior input is as given by NAM (2023).

## Appendix C Ensemble Smoother with Multiple Data Assimilation (ES-MDA)

Ensemble Smoother with Multiple Data Assimilation (ES-MDA) is a technique commonly used to update model parameters iteratively by minimizing the difference between model predictions and observed data. ES-MDA enhances this approach by allowing multiple updates, which helps refine parameter estimates over successive iterations.

Equation A12 gives the least-squares function that must be maximized (Tarantola 2005). The parameters are collected in a vector **m** and the settlement data in a vector **d**. The length of **m** is equivalent to the number of parameters, while the length of** d** corresponds to the number of data points ${N}_{d}$. **G(m)** is the operation of the forward model on the parameters **m**. The aim is to find **m** for which **G(m)** is closest to **d** while the model parameters are still close to their initial values. The initial parameters of the study are collected in **m**_**0**_, and the covariance of the parameters is contained in a matrix **C**_**m**_, while the covariance of the data is represented by **C**_**d**_.

A12 $$\begin{array}{c}J=\mathrm{exp}\left(-\frac{1}{2}{\left[{\boldsymbol{m}}-{{\boldsymbol{m}}}_{0}\right]}^{T} {{\boldsymbol{C}}}_{m}^{-1} \left[{\boldsymbol{m}}- {{\boldsymbol{m}}}_{0}\right]- \frac{1}{2} {\left[{\boldsymbol{d}}-{\boldsymbol{G}}\left({\boldsymbol{m}}\right)\right]}^{T}{{\boldsymbol{C}}}_{d}^{-1}\left[{\boldsymbol{d}}-{\boldsymbol{G}}\left({\boldsymbol{m}}\right)\right]\right) \end{array}$$

An ensemble of parameter values is collected in a vector **M** = (**m**_**1**_**, m**_**2,**_** …, m**_**Ne**_) of length ${N}_{e}$, the ensemble size. The values of **m** are taken from the prior estimate and standard deviation of the parameters. The ensemble of data vectors is created by adding random noise to the original data, again using a Monte Carlo approach, resulting in **D** = (**d**_**1**_**, d**_**2**_**, …, d**_**Ne**_).

Then, the least-squares solution of Eq. 15 is solved for the entire ensemble at once, so that **GM** replaces **G(m)** in Eq. 15. **GM’** is the difference between **GM** and its average and **M’** is the difference between **M** and its average. The covariance is defined as **C**_**m**_ = **M’M’**^**T**^/(*N*_*e*_-1). The new ensemble of parameters for a subsequent assimilation step can then be calculated with one of two equivalent expressions:

A13 $$ \begin{gathered} \hat{M} = M + M^{\prime}[GM^{\prime}]^{T} (GM^{\prime}[GM^{\prime}]^{T} + (N_{e} - 1)C_{d} )^{( - 1)} (D - GM) = \hfill \\ M + M^{\prime}([GM^{\prime}]^{T} C_{d}^{( - 1)} GM^{\prime} + (N_{e} - 1)I)^{( - 1)} [GM^{\prime}]^{T} C_{d}^{( - 1)} (D - GM) \hfill \\ \end{gathered} $$

For which $\widehat{{\boldsymbol{M}}}$ is the estimated ensemble of parameters. Depending on the number of parameters and the number of data points the expression with the smallest calculation time can be chosen.

The newly estimated parameters and standard deviation that follow from Eq. 16 can be interpreted as the final result or they can be used in a repeated optimization; this is the Multiple Data Assimilation feature. A repetitive application results in a better estimate of the parameters in the case of a non-linear forward model (Emerick and Reynolds 2013). During the repetitive application of parameter fitting, the data remains the same. To compensate for overfitting towards the multiple application of the same dataset, the covariance of the data is increased for each step of the optimization, with a factor ${\alpha }_{i}$. The set of factors is constrained by ${\sum }_{i=1}^{nI}\frac{1}{{\alpha }_{i}}=1$, with $nI$ the number of assimilation steps (Emerick and Reynolds 2013; Fokker et al. 2019; Verberne et al. 2023). When ${\alpha }_{i}$ reduces with a factor *q* with every assimilation step, the following starting value must be used:

A14 $$\begin{array}{c}{\alpha }_{0}=\frac{1- {q}^{nI}}{{q}^{nI-1}- {q}^{nI}}\end{array}$$

Ensemble collapse, the phenomenon that all the parameters converge and there is minimal to no spread of the parameter values in the ensemble of parameters, is often observed with parameter optimization studies of subsidence (e.g. Verberne et al. 2023). Inflation can be used to increase the ensemble spread of the parameter values to more realistic values, address sampling errors and counteract model error impact (e.g. Anderson 2007). An inflation is applied to the ensemble deviations of the parameter values (**M’**) before the update step of Eq. 16, thus immediately after computing the **M’**:

A15 $$ M^{\prime}_{\inf lated} = M^{\prime} \times \gamma $$

In which $\gamma \ge 1.$ The optimal $\gamma$ is determined through trial and error, starting from no inflation and step-wise increasing with 0.1.

*Performance assessment*

To assess the quality of the parameter optimization results the Absolute Error (AE) and Average Ensemble Spread (AES) are employed (Franssen and Kinzelbach, 2008). The AE indicates the difference between the data and the estimate of the data:

A16 $$\begin{array}{c}AE=\frac{1}{{N}_{e} {N}_{dd}} {\Sigma }_{j=1}^{{N}_{e}}{\Sigma }_{i=1}^{{N}_{dd}}\left|d{d}_{i.j}-d{d}_{i,true}\right|\end{array}$$

In which ${N}_{e}$ is the number of ensembles, $d{d}_{i.j}$ the estimated points and $d{d}_{i,true}$ the measured value. When the actual values of parameters are known it is possible to quantify the AE for the model parameters. As these are unknown in this study, only an estimate for the data is provided.

The AES indicates the variation of the values with respect to the average, i.e. how large the variance in the prediction is:

A17 $$\begin{array}{c}AES=\frac{1}{{N}_{e }{N}_{dd}} {\Sigma }_{j=1}^{{N}_{e}}{\Sigma }_{i=1}^{{N}_{dd}}\left|d{d}_{i,j}-\overline{d{d}_{i}}\right| \end{array}$$

To provide an assessment of the update from the prior to posterior the reduction of both the AE and AES is calculated as:

A18 $$\begin{array}{c}AES=\frac{1}{{N}_{e }{N}_{dd}} {\Sigma }_{j=1}^{{N}_{e}}{\Sigma }_{i=1}^{{N}_{dd}}\left|d{d}_{i,j}-\overline{d{d}_{i}}\right| \end{array}$$

The closer a value to zero, the stronger the constraining force of the ES-MDA. If the constraining force is too strong, the spread of the posterior estimates indicates is lower than realistic, an ensemble collapse. This makes the results unreliable for uncertainty quantification. In this study the normalized version for AE and AES as given in Eq. 11 are calculated, but for simplicity they are referred to as AE and AES.
